# Differential Effects of Blood-Flow Restriction and High-Intensity Resistance Training on Cortical Thickness and White Matter Integrity in Older Men: A Three-Group Randomized Controlled Trial

**DOI:** 10.3390/brainsci16080797

**Published:** 2026-07-28

**Authors:** Milda Butkienė, Urtė Lukoševičiūtė, Viltė Pažėraitė, Kristina Valatkevičienė, Rymantė Gleiznienė, Dalia Musneckienė, Saulius Lukoševičius, Lina Mickevičienė, Robertas Petrolis, Vida J. Česnaitienė, Oron Levin, Nerijus Masiulis, Wouter A. J. Vints

**Affiliations:** 1 Department of Radiology, Medical Academy, Lithuanian University of Health Sciences, LT-44307 Kaunas, Lithuania; urte.lukoseviciute@stud.lsmu.lt (U.L.); vilte.pazeraite@stud.lsmu.lt (V.P.); kristina.valatkeviciene@lsmu.lt (K.V.); rymante.gleizniene@lsmu.lt (R.G.); saulius.lukosevicius@lsmu.lt (S.L.); 2 Department of Neurology, Medical Academy, Lithuanian University of Health Sciences, LT-44307 Kaunas, Lithuania; dalia.musneckiene@lsmu.lt; 3 Department of Health Promotion and Rehabilitation, Lithuanian Sports University, LT-44221 Kaunas, Lithuania; lina.mickeviciene@stud.lsu.lt (L.M.); vida.cesnaitiene@lsu.lt (V.J.Č.); levin.oron@lsu.lt (O.L.); nerijus.masiulis@lsu.lt (N.M.); w.vints@maastrichtuniversity.nl (W.A.J.V.); 4 Department of Physics, Mathematics and Biophysics, Lithuanian University of Health Sciences, LT-44307 Kaunas, Lithuania; robertas.petrolis@lsmu.lt; 5 Neuroscience Institute, Lithuanian University of Health Sciences, LT-44307 Kaunas, Lithuania; 6 Department of Rehabilitation Medicine, Care and Public Health Research Institute, Maastricht University, 6200 MD Maastricht, The Netherlands; 7 Centre of Expertise in Rehabilitation and Audiology, Adelante Zorggroep, 6432 CC Hoensbroek, The Netherlands

**Keywords:** blood-flow restriction training, high-intensity resistance training, cortical thickness, white matter integrity, muscle-brain axis

## Abstract

**Objectives**: While longitudinal studies have linked parietal lobe structure and muscle mass, no resistance exercise studies have evaluated whether interventions can have a positive impact on parietal lobe structure. Therefore, we investigated the effects of high-intensity resistance training (HIRT) and blood-flow restriction training (BFRT) on cortical thickness and white matter integrity in the parietal region, as well as in other regions, in an exploratory manner. **Methods**: A total of 63 older men were assigned to BFRT, HIRT or control groups for 12 weeks of intervention. A total of 48 participants completed brain and thigh magnetic resonance imaging before and after intervention. Cortical thickness was assessed using FreeSurfer, fractional anisotropy (FA) values were calculated using ExploreDTI, thigh muscle anatomical cross-sectional area was measured from MRI. **Results**: A significant difference in cortical thickness was observed only in HIRT group in the left parietal cortex—inferior parietal, supramarginal and posterior cingulate. Exploratory analyses revealed cortical thickness changes in other left-hemisphere regions, including the temporal (bankssts, inferior temporal), frontal (medial orbitofrontal, precentral, rostral middle frontal), and occipital (lateral occipital) cortices. In contrast, the BFRT group showed significant FA value change in parietal region – in left inferior parietal, left precuneus and right superior parietal. Exploratory analyses showed FA value change in additional regions in the BFRT group, including the left frontal (caudal middle frontal and superior frontal), left temporal (fusiform), right occipital (lateral occipital), and in the HIRT group, in the left frontal region (pars opercularis). **Conclusions**: HIRT and BFRT were associated with beneficial brain structural changes, although through distinct neurobiological mechanisms, with HIRT primarily influencing cortical thickness and BFRT predominantly affecting white matter microstructure.

## 1. Introduction

The world’s population is undergoing a profound demographic shift, characterized by declining birth rates and increasing life expectancy, leading to rapid population aging and creating multifaceted public health challenges, including risk of cognitive decline [1]. With advancing age, the brain experiences various morphological changes, including shifts in neurotransmitter levels and cellular damage which are accompanied by neuroinflammation [2]. Collectively, these changes contribute to gray and white matter degeneration, cortical thinning, ventricular enlargement, and widening of the sulci, leading to overall brain volume loss and subsequent functional decline [3]. These age-related brain structural alterations critically influence memory, learning, and other cognitive functions [4]. Paralleling these cerebral changes, skeletal muscle also undergoes age-related deterioration, characterized by progressive loss of muscle mass and strength [5]. Importantly, however, converging evidence suggests that both the brain and skeletal muscle retain substantial plasticity, making them highly responsive to exercise-induced stimuli, allowing exercise-induced neuroprotective and myoprotective adaptations to occur [6,7].

In general, age-related structural changes are diffusely present across the cortex, but particular regions, such as the frontal, parietal and temporal cortices, are characterized by greater cortical thinning compared to other cortical regions [8,9]. This spatially patterned vulnerability, which follows an anterior-to-posterior gradient, was among the first systematically documented by Pfefferbaum and colleagues (1994) using volumetric magnetic resonance imaging (MRI), and has subsequently been substantiated across multiple studies, consistently demonstrating greater shrinkage in frontal compared to occipital and other posterior cortical regions [10]. There is substantial evidence that age-related cortical thinning in healthy adults has been observed in frontal, temporal, limbic, parietal and occipital regions when compared to younger adults [10,11,12,13,14]. Given the functional roles of these regions, such structural changes are thought to underlie the well-documented age-related declines in executive function, memory, and attentional processing observed in older adults [15,16]. However, a substantial body of evidence suggests that regular physical activity may slow age-related cognitive decline and help protect against brain atrophy [17].

Recent evidence indicates a link between age-related brain structural changes and changes in skeletal muscle mass. For example, longitudinal studies have shown that low muscle mass is associated with parietal lobe atrophy and cognitive decline [18,19,20]. These studies identified the parietal lobe as the cortical region most consistently associated with muscle loss, providing the rationale for selecting it as the primary region of interest in our study. Furthermore, a large-scale cross-sectional study, including middle-aged healthy adults, discovered a relationship between greater thigh muscle volume and greater brain volume [21].

Several biological pathways have been proposed to mediate communication between skeletal muscle volume and brain health. Among these, lactate has been suggested as a potential mediator of exercise-induced neuroplasticity, whereas muscle-derived factors, also known as myokines and myometabolites, have been proposed to positively impact cognitive function by exerting anti-neuroinflammatory effects [22,23]. However, the relative contribution of these mechanisms remains incompletely understood.

As mentioned earlier, while growing evidence supports the existence of a muscle–brain axis, and longitudinal studies have shown a significant association between structure of parietal lobe and muscle mass, no exercise studies have yet evaluated whether resistance training interventions increasing muscle mass can have a positive impact on parietal lobe atrophy; previous studies focused only on aerobic exercise [19,24,25]. It has been shown that aerobic exercise training was associated with improved white matter microstructural integrity in patients with amnestic mild cognitive impairment, particularly within prefrontal white matter tracts, despite the absence of significant group-level effects [26]. Therefore, in the current study we sought to examine how two different types of lower-limb exercise programs—high-intensity resistance training (HIRT) and blood-flow restriction training (BFRT)—leading to increased thigh muscle anatomical cross-sectional area (ACSA), corresponding to muscle mass, can influence structural brain changes in older adults in comparison with a control group that did not engage in any exercise. In this study, in addition to regular resistance training, we chose to include, as mentioned previously, low-load blood-flow restriction training, which has recently emerged as a relatively new promising resistance training method, which compared to regular resistance training is expected to elicit increases in muscle strength and hypertrophy while resulting in lower risk of muscle damage by applying significantly lower mechanical loads, making it particularly suitable for frail individuals who may not tolerate heavy resistance training [27,28]. Moreover, it has been reported that BFRT is associated with significant increase in lactate levels due to the high metabolic load induced by restricted muscle perfusion [29]. On the other hand, HIRT with high mechanical load is associated with little lactate response but significant muscle damage, commonly assessed by circulating creatine kinase (CK) concentrations [30]. These distinct physiological responses provided the rationale for including these parameters in the present study.

Mechanistically, skeletal muscle communicates with the brain through endocrine signaling pathways, including secretion of myokines and neurotrophic factors, thereby supporting the concept of muscle–brain crosstalk [31]. Emerging evidence indicates that BFRT may influence these pathways and represent a promising strategy to enhance muscle–brain crosstalk while minimizing load-related tissue stress [32]. We hypothesize that the beneficial effect of BFRT on both muscle and brain integrity will be higher than that achieved with resistance training alone as the former is expected to induce less inflammatory effects and maximize effect of intervention on muscle–brain crosstalk.

Given the association between muscle mass and parietal lobe atrophy found in two recent longitudinal studies, we will specifically focus on exercise-induced effects on the parietal lobe [19,33]. Accordingly, the parietal region represented the primary hypothesis-driven region of interest in the present study. To date, the specific interplay between resistance training-induced muscle adaptation and parietal cortical plasticity remains largely unexamined. However, converging evidence from motor learning and proprioceptive training studies collectively supports the plausibility of such an association [34,35]. Given that neuroplastic changes associated with exercise are not confined to the parietal cortex but have been observed throughout the brain [36,37,38], a secondary, exploratory objective of this study was to investigate whether resistance exercise elicits comparable structural and functional adaptations across additional brain regions. Therefore, findings outside the parietal cortex should be considered exploratory in nature and were intended to identify potential regions of interest for future studies.

## 2. Materials and Methods

### 2.1. Pre-Registration

This study is part of a trial named Blood-Flow Restriction and High-Intense Resistance Training in Aging: Interactions Between Neuroplasticity and Muscle (BRAIN-M), registered in ClinicalTrials.gov under Identifier number NCT05744167.

### 2.2. Participants and Setting

**Figure 1:**
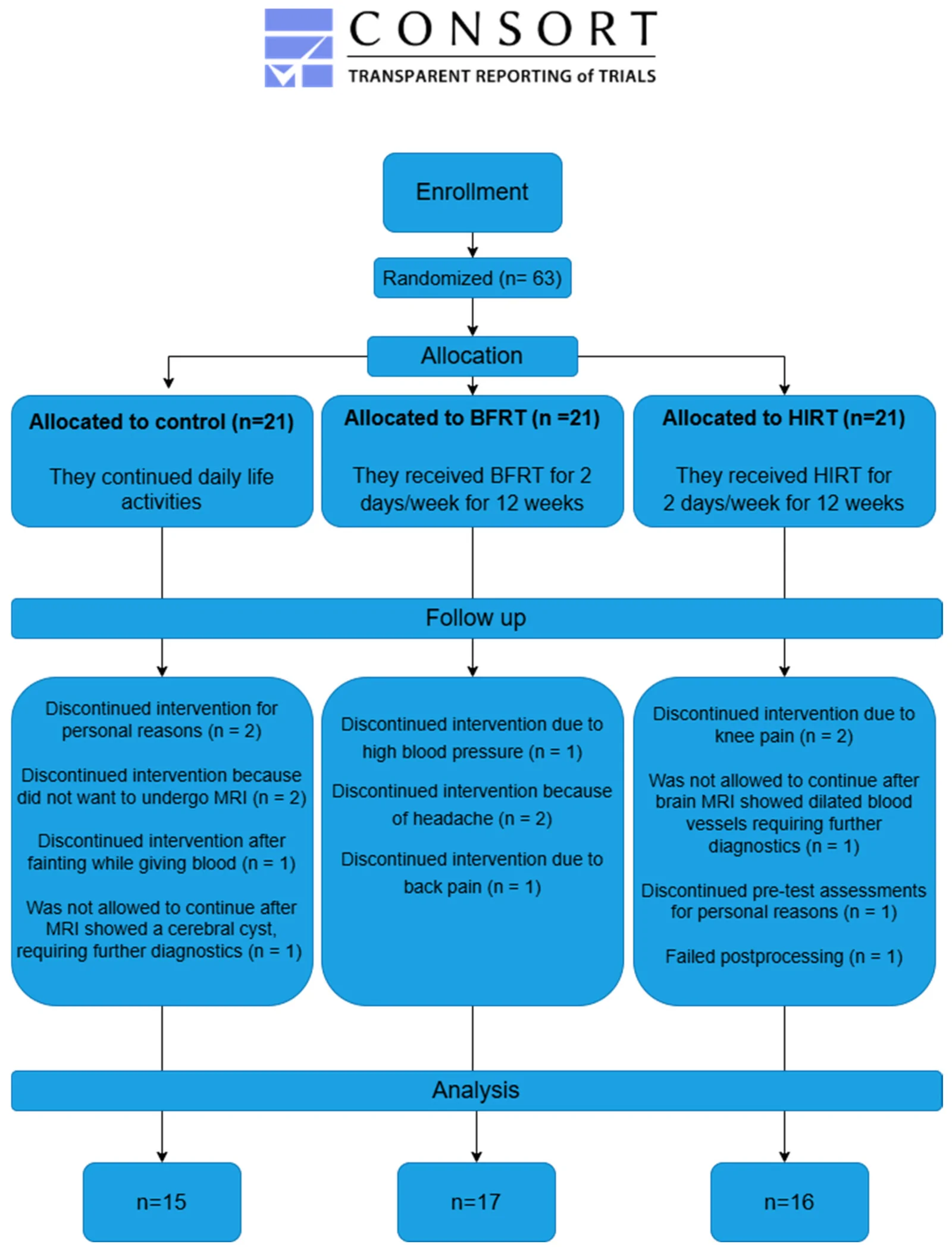
CONSORT 2010 flow diagram.

The BRAIN-M trial, conducted at the Lithuanian Sports University in Kaunas, Lithuania, enrolled sixty-three community-dwelling males aged more than 60 years between October 2022 and November 2023. Recruitment was carried out through advertisements in local and national newspapers, the university’s website, and local events for older adults. A participant flow diagram is shown in Figure 1.

Exclusion criteria, in addition to age and sex, included alcohol or drug abuse, cognitive, neurological, cardiovascular, oncological, or psychiatric conditions; prior lower extremity injury; osteoarthritis; diabetes mellitus; obesity (body mass index (BMI) exceeding 30 kg/m^2^, when it precluded safe of technically adequate MRI acquisition, according to the scanner specifications of maximum table weight of 250 kg and bore diameter of 70 cm); history of deep vein thrombosis; prior chemotherapy; or psychopharmacological drug use within the preceding five years. Moreover, participants regularly involved in an exercise program within the six months before enrolment were excluded. Participants were allowed to withdraw from the study at any time. The study methods were approved by the Kaunas Regional Biomedical Research Ethics Committee (Nr. BE-2-22) and written informed consent was obtained from all participants prior to their inclusion in the study.

### 2.3. Study Design

This exploratory pilot study was designed as a parallel, three-group randomized controlled trial which was conducted to compare two different resistance exercise interventions with a control condition. We used minimization for group allocation, a technique that maintains balance between groups based on one or more prognostic factors [39]. In the BRAIN-M trial, baseline handgrip strength (kg) was identified as a variable of significant prognostic relevance and was incorporated into the minimization method as a continuous variable, thereby minimizing differences in baseline handgrip strength between study groups. Handgrip strength is considered an important clinical marker, as low values are associated with increased neurochemical indicators of neurodegeneration, cognitive impairment, and chronic inflammation. It is also the most frequently used muscle strength marker in diagnosing sarcopenia, where, according to the European Working Group on Sarcopenia in Older People, muscle strength is the key marker, followed by muscle mass and physical performance [40,41,42,43,44]. The minimization program was built by author WV in Excel. The participants’ handgrip was measured and entered in the minimization tool by author NM, who together with the coaches during training sessions was the only one with knowledge of group allocation. The highest handgrip strength value obtained from four trials (two per hand) was used in the minimization procedure. The minimization procedure began with the first six participants being randomly allocated to one of the three groups using a random number generator that appeared in the Excel spreadsheet automatically after the handgrip strength was written down. Some participants needed to be included completely at random for the criteria used in the tool to start working. Subsequently, any new participant was included in a group based on two criteria: (1) with the chosen group allocation, the sum of the differences between mean handgrip strength in each of the groups is the lowest, and (2) the largest group did not exceed the smallest group by more than three participants. If following the first rule meant adding a participant to the largest group and therefore exceeding the smallest group by more than three participants, the second-best option according to the first rule was chosen instead. The purpose of the second rule is to avoid a potential scenario where a group with initially few participants ends up receiving all remaining participants, which could possibly result in large differences in handgrip strength in this group compared to the other two groups. Participants and coaches were informed of the group assignments; however, allocation was concealed from all individuals involved in assessments or data analysis, with the exception of lactate and rate of perceived exertion (RPE) measurements, which were conducted by the coaches. Author NM supervised the training sessions. Participants’ data were pseudonymized using a random code consisting of numbers and letters that was mentioned on all study data. The key to this code was kept in a locked cabinet together with allocation information that could only be accessed by author NM.

### 2.4. Assessment: Demographic Characteristics and Questionnaires

Participants’ age, educational level, and hand dominance were documented. Participants’ height and weight were assessed, and BMI was calculated.

The International Physical Activity Questionnaire—Short Form (IPAQ-SF) was used to estimate physical activity level. Self-reported physical activity level is measured based on the total MET minutes per week during exercise of light, moderate or vigorous intensity, using the formula: total MET-minutes/week = the sum of days performing light/moderate/vigorous activity per week × average time/day performing these activities × F, where F equals the MET level 3.3 for light-intensity exercise, 4.0 for moderate-intensity exercise and 8.0 for vigorous-intensity exercise. Participants were categorized according to the amount of MET minutes per week: <600 MET minutes per week as sedentary, 600–3000 MET minutes per week as moderately active and >3000 MET minutes per week as highly active [44].

Assessment: Strength measurements

Baseline strength was assessed by measuring the one repetition maximum (1RM) for each of the three intervention exercises, for participants allocated to one of the intervention groups as well as those allocated to the control group: leg press, leg extension and leg curl. A 5RM test was conducted, based on a standard protocol recommended by the National Strength and Conditioning Association [45]. The estimated 1RM was computed using the ExRx.net calculator (https://exrx.net/Calculators/OneRepMax, accessed on 20 April 2024) [46]. Once every four weeks, the 5RM test was repeated for subjects in one of the two intervention groups and repeated for all three groups at the end of the twelve-week program.

Handgrip strength (in kg) was measured using an adjustable handgrip strength testing system (JAMAR 11940248) in standing position. As described in the manual, the size of the grip was selected such that subjects may be able to hold the dynamometer with a 90° angle in the second joint of the index finger. Before the real test, one try at submaximal effort was allowed. After this, the participants repeated the test two times at maximal effort following 1 min interval between trials for both hands. Among the measurements, the highest value was used.

Assessment: Blood sampling

Baseline and post-exercise lactate and CK levels were measured immediately after the first exercise bout for lactate and 48 h after the first training session following increasing of the training load (every 4 weeks of training). Lactate was measured with the Arkray Lactate Pro 2 from a drop of blood collected from the fingertip. CK was measured from whole blood collected from the antecubital vein in an EDTA tube.

### 2.5. Magnetic Resonance Imaging

All participants underwent both brain and thigh MRI at the baseline and after three months in a 3 Tesla Skyra scanner (Siemens Healthineers, Erlangen, Germany) with a 32-channel receiver head coil and 2 16-channel receiver extremity coils. The total duration of examination lasted approximately 90 min.

#### 2.5.1. Brain Imaging and Volumetric Analysis

All MRI datasets underwent visual inspection for image artifacts and excessive motion before processing. No datasets were excluded because sequences affected by motion artifacts were repeated during the MRI examination. Cortical thickness was measured at the baseline and after three months using a three-dimensional high-resolution T1-weighted Magnetization Prepared Rapid Gradient Echo (MPRAGE) sequence with the following parameters, repetition time (TR) =  2200 ms, echo time (TE) =  2.48 ms, 0.9  ×  0.9  ×  1.0 mm^3^ voxel size, field of view (FOV): 230  ×  256 mm, and number of sagittal slices =  176, and co-localized T2-weighted SPACE Dark Fluid sequence with the following parameters: TR =  7000 ms, TE =  394 ms, inversion time =  2100 ms, FOV =  192  ×  256 mm, number of sagittal slices =  176, and slice thickness of 1 mm. Cortical surface area and thickness maps were automatically created according to the Destrieux atlas using the longitudinal pipeline of FreeSurfer (FreeSurfer v7.1.1, Harvard, MA, USA, http://surfer.nmr.mgh.harvard.edu/, accessed on 17 November 2024) [47]. The quality control of the cortical reconstruction and segmentation was assessed by visual inspection of all Freesurfer outputs, including skull stripping, white matter segmentation, pial surface placement, and cortical parcellation. When inaccuracies were identified, minor manual corrections were performed in 12 of 96 processed datasets (including pre- and post-intervention) and primarily involved adjustments to skull stripping and white matter segmentation using quality assurance tools implemented in FreeSurfer (https://surfer.nmr.mgh.harvard.edu/fswiki/QATools, accessed on 17 November 2024). The affected datasets were reprocessed, and the corrected outputs underwent a second visual quality assessment prior to inclusion in the analyses. There was one dataset in the HIRT group which was excluded due to unsuccessful cortical reconstruction and segmentation despite repeated processing and manual correction attempts. The analysis was blinded to the group and the assessment time.

#### 2.5.2. Brain Imaging and Regional Fractional Anisotropy Analysis

Whole-brain diffusion tensor imaging (DTI) data were acquired using a spin-echo EPI sequence ep2d_diff_DTI_dir with the following parameters: 76 slices, 64 diffusion directions (b = 1000 s/mm^2^, averages 1; b = 0 s/mm^2^, averages 12), 2 interleaved volumes without diffusion weighting (b = 0 s/mm^2^; b = 1000 s/mm^2^), voxel size = 1.7 × 1.7 × 2.0 mm^3^, TE/TR = 78.0/7100 ms, and matrix size = 122 × 128.

DTI data were analyzed using ExploreDTI software v.4.8.6 [48]. Postprocessing steps included image conversion from DICOM to NIFTI, conversion of b-values and -vector to B-matrix, correction for signal drift, Gibbs ringing, subject motion and EPI distortion. The whole-brain analysis encompassed 179 brain regions, 109 cortical and 70 white matter regions, based on the FS_cvs_avg35_inMNI152 FreeSurfer template [49]. Variable of interest was fractional anisotropy (FA). All datasets subsequently underwent visual quality assessment for residual artifacts and image quality before tensor estimation and statistical analyses. Quality-control procedures were performed using identical processing parameters for all participants and data sets. The analysis was blinded to the group and the assessment time.

#### 2.5.3. Thigh Imaging

Bilateral axial MRI scans of the thighs were performed from iliac crests to the articular surface of the tibia. The thigh MRI was conducted immediately after the brain MRI with subjects lying supine to prevent fluid shift from changing positions [50]. To avoid movement and reduce compression of the legs, their heels were secured on a non-metallic support. Axial scans of the thighs were obtained using a T2-weighted Dixon sequence, with the following scanning parameters: TR =  5810 ms, TE =  93 ms, FOV =  400 × 462 mm, and a slice thickness of 5 mm.

**Figure 2:**
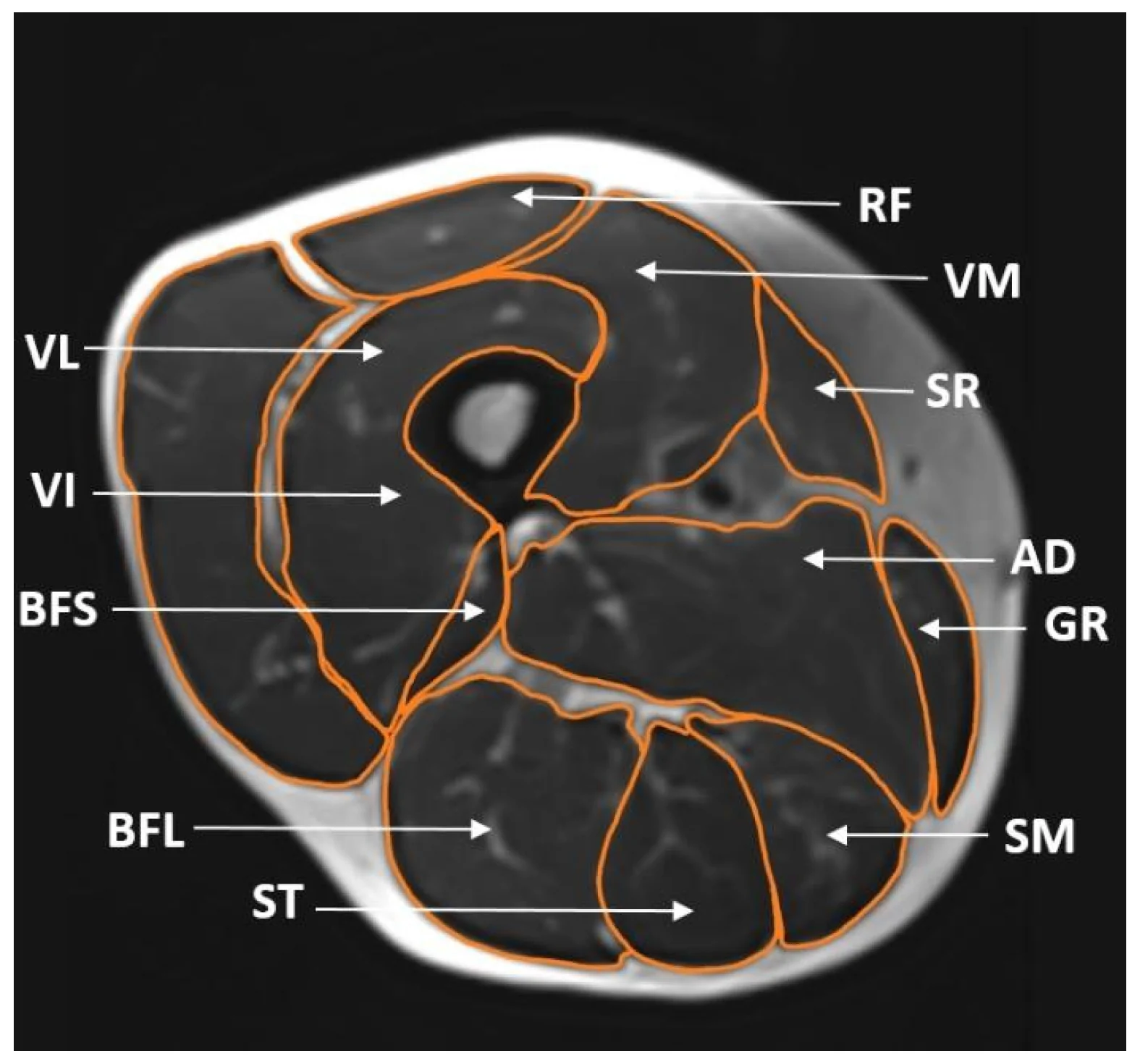
Cross-sectional area of the muscles of the right thigh at 50% of the femur length. Abbreviations: rectus femoris (RF), vastus lateralis (VL), vastus intermedius (VI), and vastus medialis (VM), biceps femoris short head (BFS), biceps femoris long head (BFL), semitendinosus (ST), semimembranosus (SM), adductor longus, brevis, magnus and pectineus together (AD), gracilis (GR), and the sartorius (SR).

Thigh muscle ACSA was measured at 50% of femur length (the distal site was obtained as 0%). Femur length was defined as a distance between the top of the greater trochanter and the bottom of the lateral condyle. Only the right thigh was analyzed in all participants to ensure a standardized assessment protocol. Muscles of the right femur were manually segmented as shown in Figure 2. Measurements of the femur for each subject were done manually by two experienced radiologists using integrated MedDream image viewing and measurement software. The radiologists were blinded to the participant group allocation and assessment time. By adding all the muscle ACSAs, an approximation of the whole thigh ACSA was acquired. Visible intermuscular fat, blood vessels, nerves and femur bone were eliminated as extensively as possible. ACSA measurements were repeated by the same radiologist in a random subset of 12 participants, demonstrating excellent intra-rater reliability (ICC = 0.996).

The muscles selected for segmentation were the four heads of the quadriceps femoris (rectus femoris (RF), vastus lateralis (VL), vastus intermedius (VI), vastus medialis (VM)), the four muscles composing the hamstring muscles (biceps femoris short head (BFS), biceps femoris long head (BFL), semitendinosus (ST) and semimembranosus (SM)), and adductors (adductor longus, brevis, magnus and pectineus together (AD), gracilis (GR), sartorius (SR)).

### 2.6. Intervention: Resistance Exercise Intervention

**Figure 3:**
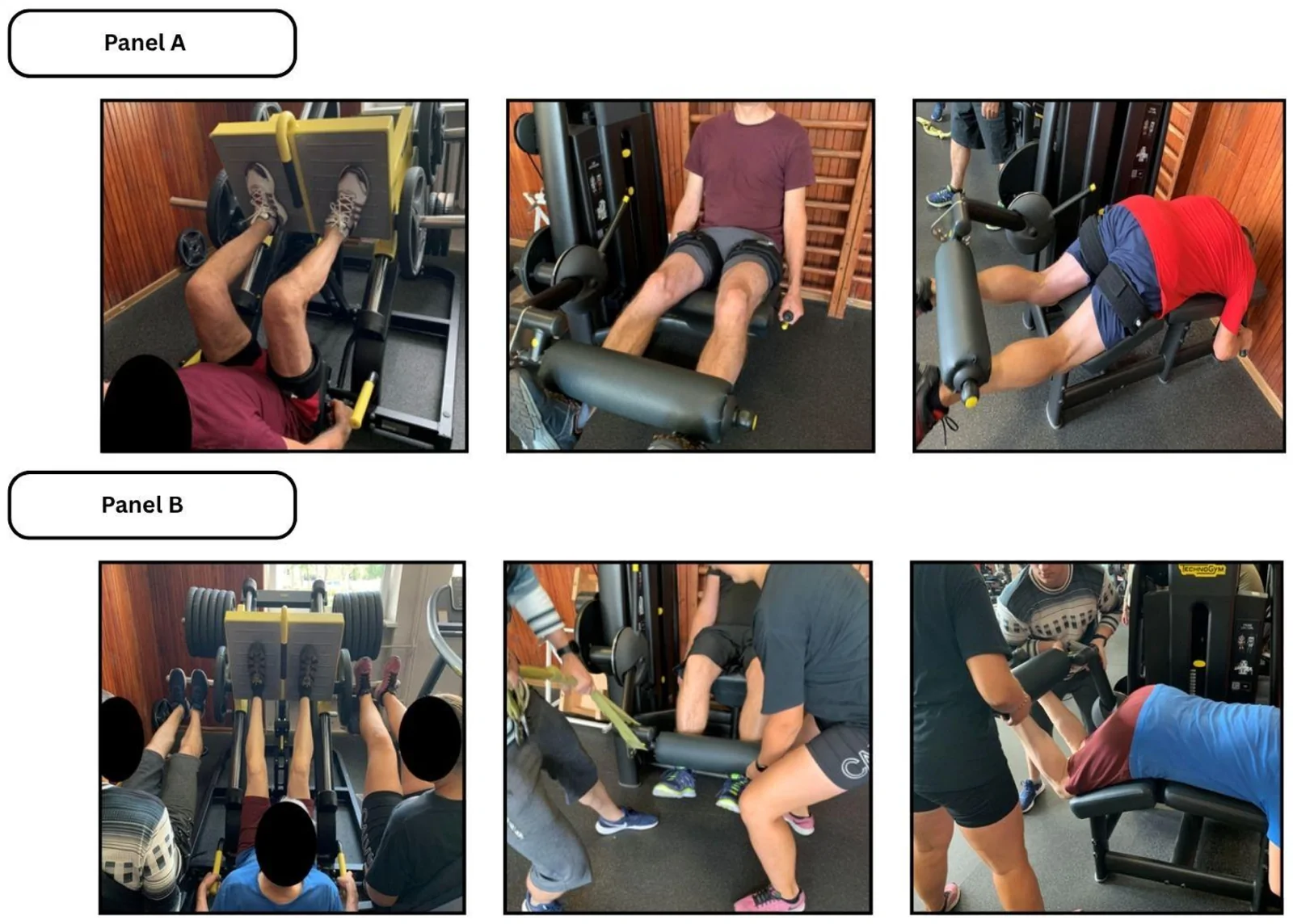
Overview of exercises. Panel (**A**) demonstrates the eccentric-only condition, in which the concentric phase of exercise was assisted by two coaches. Panel (**B**) demonstrates the blood-flow restriction training.

The study protocol is summarized in Table 1 and Table 2. The subjects in the intervention groups exercised twice a week for twelve weeks. Before each session a 10 min warm-up was done on veloergometer at a workload (W) equal to body weight (kg) and 60–80 revolutions per minute, followed by 40–50 min resistance exercises that included three lower-limb exercises—leg press, leg extension and leg curl with either BFRT or HIRT. Figure 3 illustratively demonstrates the features and progression of both interventions. The training protocols were matched for relative training volume (V% = repetitions × sets × exercise × %1RM), which equaled 57.6%. BFRT was carried out at 40% of 1RM intensity, using occlusive cuffs placed at the proximal thighs to restrict venous blood flow. Blood-flow restriction was applied using the SmartCuffs 3.0 PRO Elite system (Smart Tools Plus LLC, Strongsville, OH, USA). Four-inch (10.16 cm)-wide pneumatic cuffs were placed proximally on both thighs. Individual arterial limb occlusion pressure (ALOP) was determined automatically by the device before training. Training pressure was individualized for each participant, starting at 50% of ALOP during the first week and progressively increasing by 10% each week until reaching 80% of ALOP in week 4, which was then maintained for the remainder of the intervention. Resting blood pressure was measured before participation in the study. Individuals with a resting systolic blood pressure > 150 mmHg were not eligible for participation. Stopping criteria for BFRT were based on participants’ subjective tolerance to the intervention. Training was discontinued if participants reported symptoms such as headache, dizziness, or marked weakness.

**Table 1:** Exercise protocols. 1RM tests were carried out on rest days later in the week and the load was modified accordingly starting from the following week. The final 1RM assessment took place during the week of the last training session or the subsequent week. Abbreviations: 1RM, one-repetition maximum test; BFRT, blood-flow restriction training; RT, traditional resistance exercise training; EOE, eccentric-only exercise.

Protocol	Intensity (% 1RM)	Number of Exercises	Sets per Exercise	Rest Between Sets	Rest Between Exercises	Volume (%)
BFRT: 1 × (12r − 12r − 12r − 12r)/exercise	40%	3	4	30 s	3 min	57.6
Conc: 1 × (6r − 6r − 6r − 6r)/exercise	80%	3	4	2 min	3 min	57.6
Ecc: 1 × (4r − 4r − 4r − 4r)/exercise	120%	3	4	2 min	3 min	57.6
Week	BFRT group			HIRT group		
Week −1	1RM test			1RM test		
Week 00	Blood sampling + BFRT session			Blood sampling + EOE session		
Week 01	BFRT − BFRT			RT − RT		
Week 02	BFRT − BFRT			EOE − RT		
Week 03	BFRT − BFRT + 1RM test			RT − RT + 1RM test		
Week 04	BFRT − BFRT + Blood sampling			EOE − RT + Blood sampling		
Week 05	BFRT − BFRT			RT − RT		
Week 06	BFRT − BFRT			EOE − RT		
Week 07	BFRT − BFRT + 1RM test			RT − RT + 1RM test		
Week 08	BFRT − BFRT + Blood sampling			EOE − RT + Blood sampling		
Week 09	BFRT − BFRT			RT − RT		
Week 10	BFRT − BFRT			EOE − RT		
Week 11	BFRT − BFRT			RT − RT		
Week 12	BFRT + Blood sampling + 1RM test			EOE + Blood sampling + 1RM test		

**Table 2:** Study outline.

**Baseline**	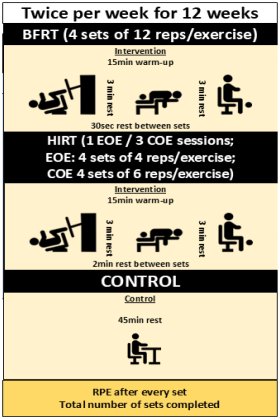	**After Completion of the 12-Week Intervention**
Handgrip strength and group allocation Questionnaires Body weight and height 1RM test MRI examination of brain and thigh		MRI examination of brain and thigh

Each repetition took 1–2 s under tension. BFRT included four sets of 12 repetitions, with 30 s of rest between sets and 3 min of rest between exercises. Within the 3 min rest, subjects were free to deflate the occlusion cuffs. HIRT was performed as either accentuated eccentric-only exercise (EOE) at 120% of 1RM or traditional resistance exercise training (RT) at 80% of 1RM, which were performed in a 1:3 ratio.

During EOE, the concentric phase of the movement was fully supported by two coaches. The eccentric phase of the movement was amplified by prolonging the time under tension to 5 s per repetition. Four sets of four repetitions with 2 min rest between sets and 3 min rest between exercises were included in each exercise.

While performing traditional RT, both the concentric and eccentric phase were completed without coaching or assistance (Figure 3). Each repetition was performed with 1–2 s under tension. The exercise included four sets of six repetitions, with 2 min rest periods between sets and 3 min breaks between exercises. Alternating between EOE and traditional RT has been reported to minimize the repeated-bout effect, resulting in inducing muscle damage during and following each EOE session [51]. This may reflect the physical strain caused by occasional daily life activities (e.g., gardening a few times a year) in untrained older adults.

Upon completion of each set of the three exercises (a total of twelve times per training session), participants reported their RPE using a 10-point Borg scale, where higher scores reflected greater experienced effort [52].

The control group did not participate in any supervised intervention or monitoring and were instructed not to change their habitual activities during the 12-week period.

### 2.7. Statistical Analysis

The data were analyzed using the statistical software IBM SPSS Statistics 30. All statistical analyses were performed using a complete-case approach. Only participants with both baseline and post-intervention MRI examinations were included in the analyses. No imputation of missing outcome data was performed because the primary outcome measures were derived from post-intervention MRI examinations that were unavailable for participants who withdrew from the study. For each participant, change scores (Δ) were calculated as the difference between post-intervention and pre-intervention measurements. Because the change scores were not normally distributed within the study groups, between-group comparisons were performed using the non-parametric Kruskal–Wallis test with pairwise post hoc comparisons. Given the skewness of the analyzed data and the presence of potential outliers, non-parametric methods were considered more appropriate. When significant overall group differences were identified, post hoc pairwise comparisons were conducted using pairwise rank comparisons with Bonferroni correction. The parietal cortex was defined a priori as the primary region of interest based on previous evidence demonstrating its sensitivity to exercise-induced structural and functional adaptations. Accordingly, analyses of the parietal region constituted the primary hypothesis-driven analyses. Individual parietal subregions and corresponding white matter tracts were evaluated as anatomically related components of this predefined hypothesis rather than as independent confirmatory hypotheses. Therefore, multiplicity correction was not applied within this primary hypothesis-testing family. Brain structural changes in the parietal region were compared between groups, as was the relationship between these changes and changes in thigh muscle ACSA over time; they were evaluated as the primary outcomes. Statistical significance for the primary hypothesis-driven analyses was declared at *p*-value < 0.05 without correction for multiple comparisons. Further, similar analyses of all remaining brain regions, including frontal, temporal and occipital regions, were considered secondary exploratory aimed at identifying potential exercise-responsive brain regions. To account for multiple comparisons, the Benjamini–Hochberg false discovery rate (BH-FDR) procedure was applied separately within each family of exploratory analyses. Specifically, BH-FDR correction was performed for (1) cortical thickness analyses (Table 5), (2) white matter FA analyses (Table 7), (3) correlations between cortical thickness and thigh muscle ACSA (Figure 7, Table 6), and (4) correlations between FA and thigh muscle ACSA (Figure 10, Table 8). A significant *p*-value that did not remain significant after FDR correction was reported as a trend.

## 3. Results

### 3.1. Participant Characteristics

A total of 63 older men (age range: 60–78) with a BMI ranging between 21.3 kg/m^2^ and 32.9 kg/m^2^ and handgrip strength ranging between 34 and 60 kg were included. Table 3 includes the baseline characteristics. There were no baseline differences between groups. Interestingly, marking differences in training effect on the first exercise bout, lactate increased more in the BFRT group (*p* = 0.010), while CK levels increased more in the HIRT group (NS).

Participants dropped out for various reasons (see Figure 1), resulting in 15, 17 and 16 participants completing measurements after 12 weeks in the control, BFRT group and HIRT group, respectively. In training groups, drop-out reasons were in part due to training-related causes, including withdrawal from the BFRT group due to increased blood pressure following training (*n* = 1), headache (*n* = 2), or inability to continue training due to back pain (*n* = 1). Training-related withdrawal from the HIRT group was due to knee pain (*n* = 2).

**Table 3:** Baseline characteristics and blood sampling results.

	BFRT (*n* = 21)	HIRT (*n* = 21)	Control (*n* = 21)	Total (*n* = 63)	*p*-Value	Missing
Age	67.19 (5.17)	66.71 (3.44)	65.05 (4.47)	66.31 (4.44)	0.264	0
Handgrip strength (kg)	48.07 (5.59)	47.79 (6.03)	47.64 (5.24)	47.83 (5.54)	0.969	0
BMI (kg/m^2^)	26.40 (2.88)	26.40 (3.01)	26.22 (2.92)	26.34 (2.89)	0.384	0
Educational level					0.971	2
Higher education	18 (85.71%)	19 (90.48%)	16 (84.21%)	53 (86.89%)		
Secondary education	1 (4.76%)	1 (4.76%)	1 (5.26%)	3 (4.92%)		
Basic education	2 (9.52%)	1 (4.76%)	2 (10.53%)	5 (8.20%)		
IPAQ MET minutes/week	5816.50 (5252.02)	5678.71 (6253.97)	4684.50 (4216.62)	5422.10 (5289.67)	0.781	4
Physical activity level					0.639	4
Sedentary	0 (0%)	1 (4.76%)	1 (5.56%)	2 (3.39%)		
Moderately active	7 (35%)	10 (47.61%)	9 (50.00%)	26 (44.07%)		
Highly active	13 (65%)	10 (47.61%)	8 (44.44%)	31 (52.54%)		
1RM leg extension (kg)	74.36 (15.94)	69.70 (17.69)	69.71 (17.83)	71.31 (17.02)	0.605	2
1RM leg curl (kg)	44.14 (9.25)	44.22 (10.08)	47.76 (15.39)	45.41 (11.87)	0.541	2
1RM leg press (kg)	180.85 (38.29)	195.86 (45.89)	193.29 (54.17)	189.81 (46.29)	0.548	2
Baseline lactate levels (mmol/L)	2.22 (1.06)	2.14 (1.09)	2.10 (0.95)	2.16 (1.02)	0.930	5
Lactate levels after first session	7.08 (2.81)	4.55 (3.09)	-	5.88 (3.18)	0.010 *	5
Baseline CK levels (μkat/L)	1.77 (1.01)	1.71 (0.99)	2.19 (1.36)	1.88 (1.12)	0.384	7
CK levels 48 h after first session (μkat/L)	4.47 (3.20)	8.16 (10.63)	-	6.22 (7.79)	0.173	8
Mean CK levels 48 h after w4, w8, w12 (μkat/L)	2.47 (1.65)	3.10 (2.41)	-	2.78 (2.05)	0.362	11
-after increasing Load w4	2.76 (2.23)	3.22 (4.23)	-	3.00 (3.34)		
-after increasing Load w8	2.48 (1.73)	3.95 (3.35)	-	3.17 (2.68)		
-after last training Session w12	2.29 (1.87)	2.61 (1.78)	-	2.45 (1.81)		

Values are presented as mean (SD) or *n* (%). For 1RM values only baseline values of participants with post-intervention values are reported. Abbreviations: 1RM, one-repetition maximum; BFRT, blood-flow restriction training; BMI, body mass index; HIRT, high-intensity resistance training; IPAQ, International Physical Activity Questionnaire; CK, creatine kinase. * Indicates statistical significance.

### 3.2. Changes in ACSA of Thigh Muscle

**Figure 4:**
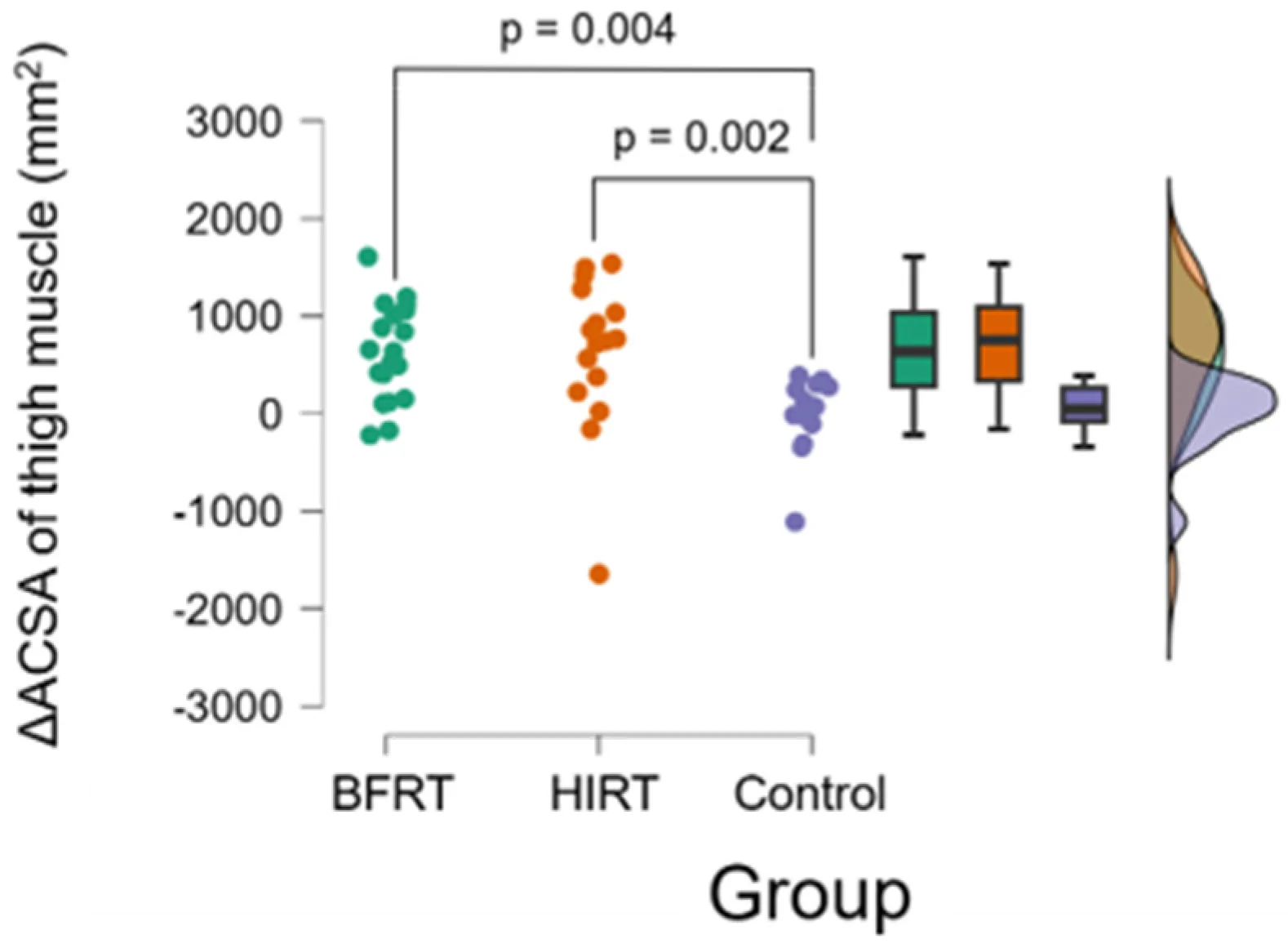
Individual data points and box plots, and violin plots of the change in thigh muscle ACSA after 12-week training. Abbreviations: ACSA, anatomical cross-sectional area; HIRT, high-intensity resistance training; BFRT, blood-flow restriction training. Individual data points represent single participants (BFRT, *n* = 17; HIRT, *n* = 16; Control, *n* = 15). Box plots display the median and interquartile range, with whiskers representing the minimum and maximum values. Violin plots illustrate the distribution density of the data.

Table 4 summarizes the changes in total thigh muscle ACSA following the 12-week intervention period; for illustration, see Figure 4. The Kruskal–Wallis test revealed a statistically significant main group effect (*p* < 0.001). Post hoc comparisons further demonstrated that both the BFRT and HIRT groups exhibited statistically significant increase in total thigh muscle ACSA, with values differing significantly from the control group (both *p* ≤ 0.004; Figure 4). However, no statistically significant difference was found in ACSA changes between BFRT and HIRT groups (*p* > 0.05; Table 4).

**Table 4:** Comparison of median change in thigh muscle anatomical cross-sectional in the three study groups.

	BFRT (Median [95% CI])	HIRT (Median [95% CI])	Control (Median [95% CI])	*p*-Value
ΔACSA mm^2^	636.40 (150.700; 1055.600)	753.90 (259.300; 1217.000)	43.15 (−156.825; 281.000)	*p* < 0.001
Post hoc comparisons between the groups	BFRT vs. HIRT			*p* > 0.05 (1.000)
	BFRT vs. Control			*p* = 0.004
	HIRT vs. Control			*p* = 0.002

### 3.3. Gray Matter Thickness Changes

**Figure 5:**
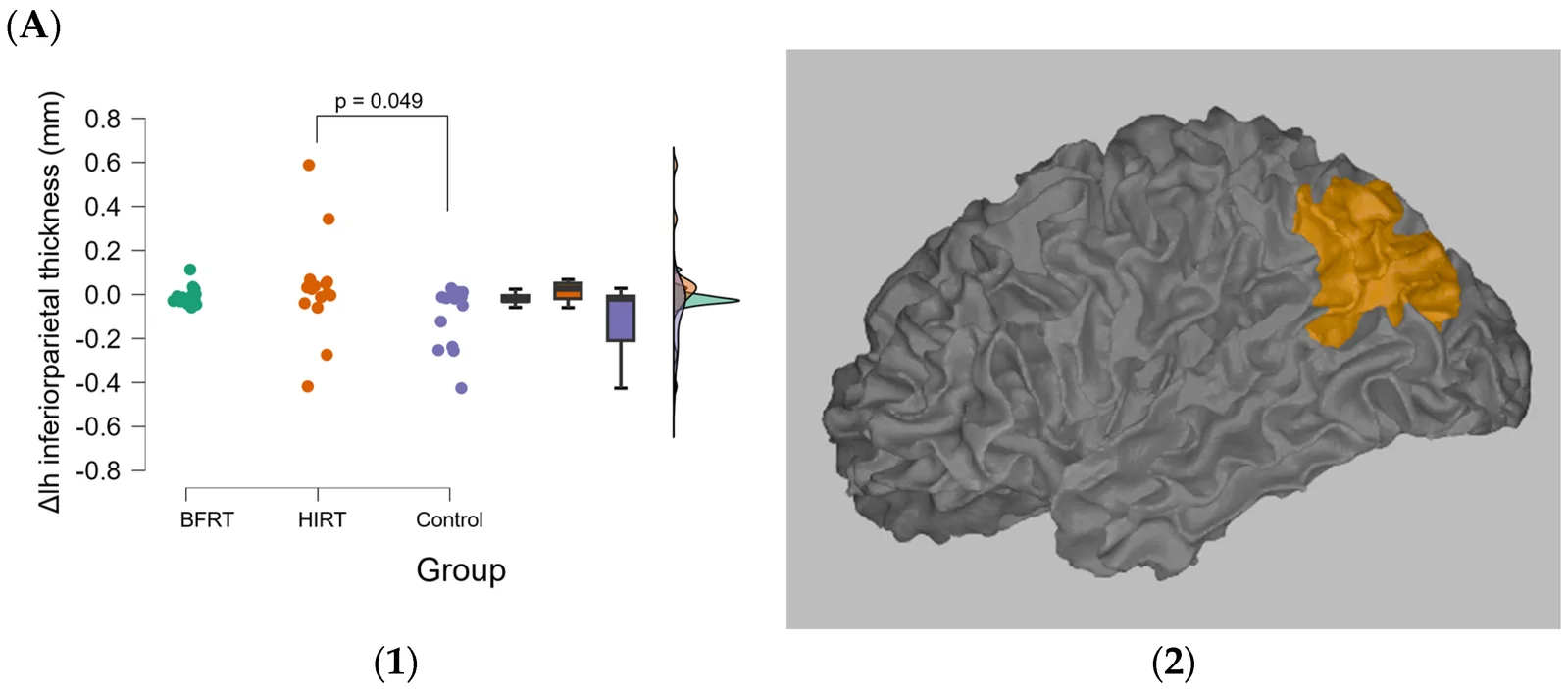

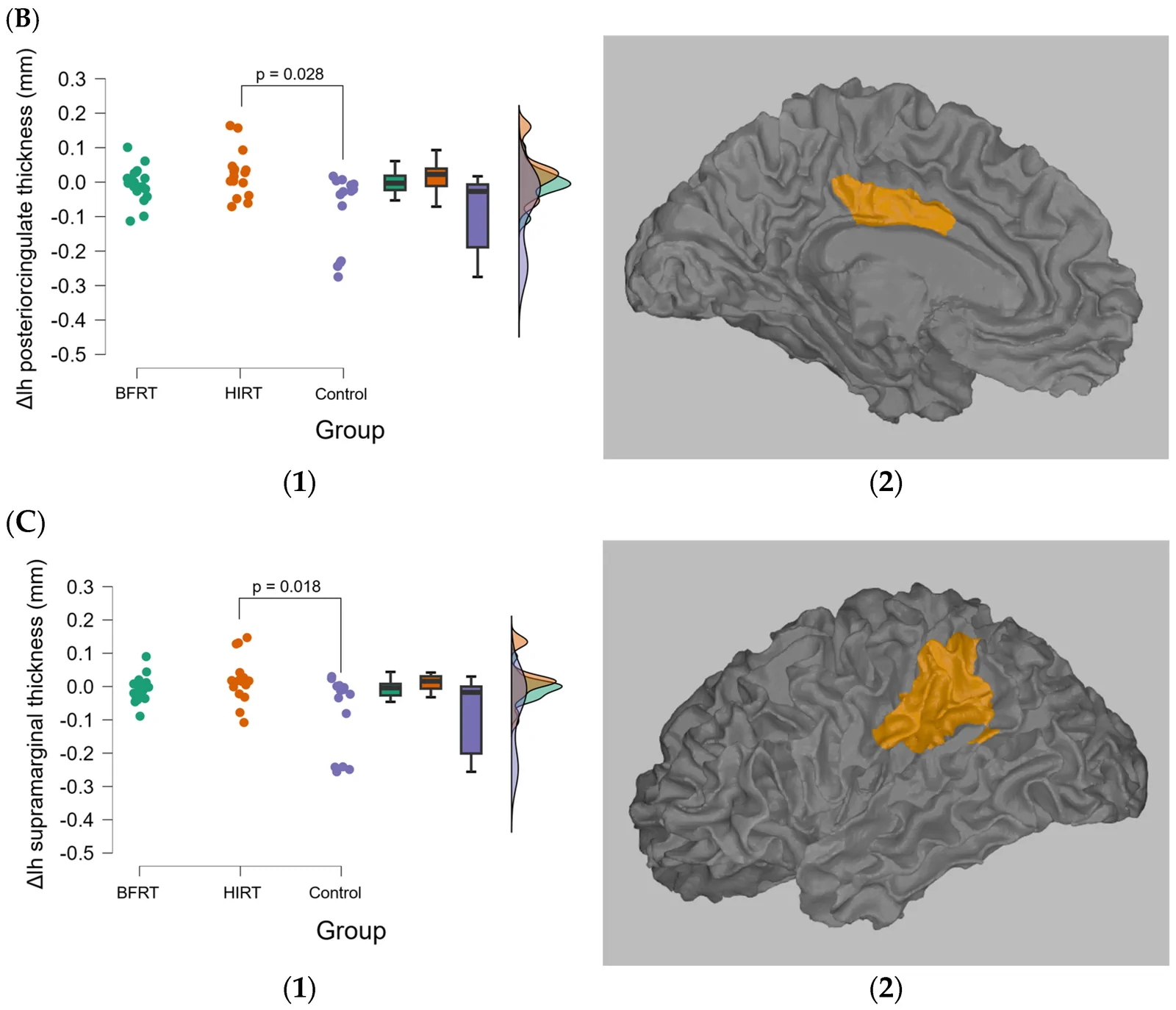
Individual data points and box plots, and violin plots (**1**) of the change in parietal cortical thickness with corresponding anatomical regions (**2**) in inferior parietal cortex (**A**), posterior cingulate cortex (**B**) and supramarginal cortex (**C**). Individual data points represent single participants (BFRT, *n* = 17; HIRT, *n* = 16; Control, *n* = 15). Box plots display the median and interquartile range, with whiskers representing the minimum and maximum values. Violin plots illustrate the distribution density of the data. Abbreviations: lh, left; HIRT, high-intensity resistance training; BFRT, blood-flow restriction training. Brain regions are displayed on the FreeSurfer template according to the Destrieux cortical atlas; sagittal projections; regions in the left hemisphere are displayed. Orange indicates regions demonstrating significant between-group differences. (**A2**) left lateral view; (**B2**) left medial view; (**C2**) left lateral view.

Table 5 summarizes the changes in cortical thickness across all ROIs (regions of interest) where a significant main effect for group was observed following the 12-week intervention period (Kruskal–Wallis tests: all *p*s ≤ 0.045). A between-group post hoc analysis revealed a significant difference between the HIRT and control groups only for cortical thicknesses of parietal lobe of ROIs in the left hemisphere. The parietal cortical regions demonstrating these changes were the left inferior parietal cortex (*p* = 0.049), left posterior cingulate cortex (*p* = 0.028) and left supramarginal cortex (*p* = 0.018); for illustration, see Figure 5. The BFRT group did not demonstrate any significant changes in parietal cortical thickness in comparison to the control group (Table 5).

**Figure 6:**
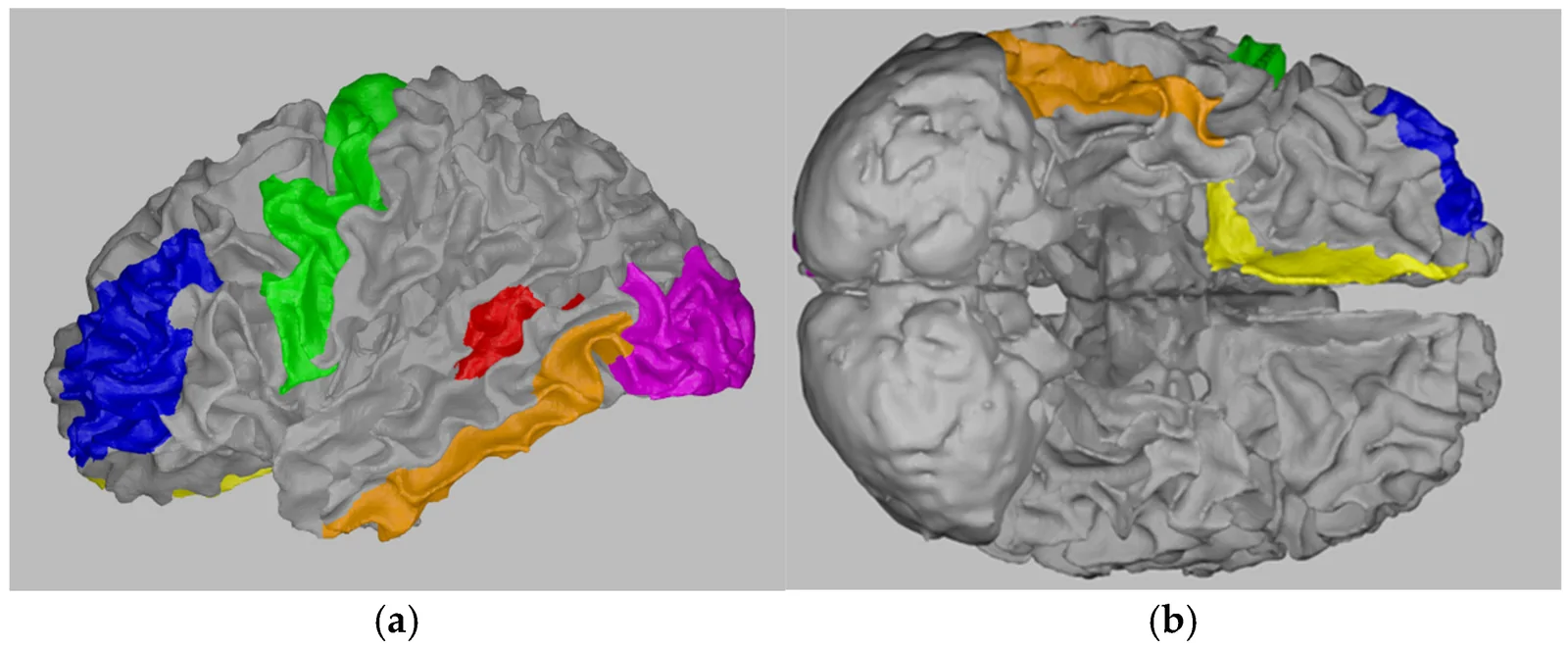
Corresponding non-parietal brain regions with significant thickness change in BFRT vs. HIRT, BFRT vs. Control and HIRT vs. Control groups. Left hemisphere ((**a**) lateral view, (**b**) bottom view), red—bankssts, orange—inferior temporal, yellow—medial orbitofrontal, green—precentral, blue—rostral middle frontal, purple—lateral occipital. Brain regions are displayed on the FreeSurfer template according to the Destrieux cortical atlas.

Similarly, nominally significant cortical thickness changes in other regions were exclusive to the HIRT group compared to controls, and all localized to the left hemisphere, including: the temporal cortex (bankssts and inferior temporal lobe; both *p*s ≤ 0.019), frontal cortex (medial orbitofrontal, precentral, rostral middle frontal; all *p*s ≤ 0.023), and occipital cortex (lateral occipital). Additionally, left medial orbitofrontal and rostral middle frontal thickness increases were nominally significantly greater in the HIRT than BFRT group (both *p* ≤ 0.015). Full results are presented in Table 5 and Supplementary Materials Figure S1. Corresponding brain parts are shown in Figure 6.

**Table 5:** Cortical thickness changes and between-group comparisons in the three study groups.

Parietal Brain Regions	BFRT, Median (IQR)	HIRT, Median (IQR)	Control, Median (IQR)	H	df	*p*-Value	BH–FDR *q*-Value
lh inferior parietal Δthickness (mm)	−0.025 (−0.031; −0.002)	0.027 (−0.033; 0.055)	−0.018 (−0.242; −0.003)	6.206	2	***p*** **< 0.05 (0.045)**	Not applicable (primary analysis)
Post hoc comparisons between the groups	BFRT vs. HIRT					*p* > 0.05 (0.212)	
	BFRT vs. Control					*p* > 0.05 (1.00)	
	HIRT vs. Control					*p* < 0.05 (0.049)	
lh supramarginal Δthickness (mm)	−0.004 (−0.033; 0.008)	0.016 (−0.017; 0.038)	−0.0175 (−0.241; 0.001)	7.695	2	***p*** **< 0.05 (0.022)**	Not applicable (primary analysis)
Post hoc comparisons between the groups	BFRT vs. HIRT					*p* > 0.05 (0.317)	
	BFRT vs. Control					*p* > 0.05 (0.576)	
	HIRT vs. Control					*p* < 0.05 (0.018)	
lh posterior cingulate Δthickness (mm)	−0.003 (−0.026; 0.026)	0.0225 (−0.046; 0.044)	−0.027 (−0.23; −0.004)	7.088	2	***p*** **< 0.05 (0.03)**	Not applicable (primary analysis)
Post hoc comparisons between the groups	BFRT vs. HIRT					*p* > 0.05 (1.00)	
	BFRT vs. Control					*p* > 0.05 (0.189)	
	HIRT vs. Control					*p* < 0.05 (0.028)	
Other non-parietal brain regions							
lh lateral occipital Δthickness (mm)	−0.006 (−0.034; 0.028)	0.0095 (−0.004; 0.048)	−0.0245 (−0.224; −0.006)	8.349	2	***p*** **< 0.05 (0.016)**	0.226
Post hoc comparisons between the groups	BFRT vs. HIRT					*p* > 0.05 (0.546)	
	BFRT vs. Control					*p* > 0.05 (0.263)	
	HIRT vs. Control					*p* < 0.05 (0.012)	
lh medial orbitofrontal Δthickness (mm)	−0.053 (−0.073; 0.004)	0.007 (−0.012; 0.0318)	−0.0135 (−0.212; −0.001)	9.570	2	***p*** **< 0.05 (0.009)**	0.226
Post hoc comparisons between the groups	BFRT vs. HIRT					*p* < 0.05 (0.012)	
	BFRT vs. Control					*p* > 0.05 (1.00)	
	HIRT vs. Control					*p* < 0.05 (0.049)	
lh precentral Δthickness (mm)	−0.014 (−0.066; 0.059)	0.007 (−0.052; 0.054)	−0.072 (−0.233; −0.017)	8.044	2	***p*** **< 0.05 (0.019)**	0.226
Post hoc comparisons between the groups	BFRT vs. HIRT					*p* > 0.05 (1.00)	
	BFRT vs. Control					*p* > 0.05 (0.075)	
	HIRT vs. Control					*p* < 0.05 (0.023)	
lh rostral middle frontal Δthickness (mm)	−0.024 (−0.053; 0)	0.026 (−0.01; 0.088)	−0.014 (−0.225; 0)	11.314	2	***p*** **< 0.05 (0.004)**	0.226
Post hoc comparisons between the groups	BFRT vs. HIRT					*p* < 0.05 (0.015)	
	BFRT vs. Control					*p* > 0.05 (1.00)	
	HIRT vs. Control					*p* < 0.05 (0.008)	
lh bankssts Δthickness (mm)	−0.012 (−0.031; 0.017)	−0.0005 (−0.021; 0.105)	−0.035 (−0.219; 0.005)	7.795	2	***p*** **< 0.05 (0.019)**	0.226
Post hoc comparisons between the groups	BFRT vs. HIRT					*p* > 0.05 (0.987)	
	BFRT vs. Control					*p* > 0.05 (0.159)	
	HIRT vs. Control					*p* < 0.05 (0.017)	
lh inferior temporal Δthickness (mm)	−0.045 (−0.073; −0.004)	−0.002 (−0.017; 0.087)	−0.0375 (−0.266; −0.009)	7.543	2	***p*** **< 0.05 (0.024)**	0.226
Post hoc comparisons between the groups	BFRT vs. HIRT					*p* > 0.05 (0.132)	
	BFRT vs. Control					*p* > 0.05 (1.00)	
	HIRT vs. Control					*p* < 0.05 (0.027)	

Abbreviations: lh, left; rh, right; HIRT, high-intensity resistance training; BFRT, blood-flow restriction training; BH–FDR, Benjamini–Hochberg false discovery rate. In the *p*-value column, bold values indicate statistically significant differences (*p* < 0.05) across all three groups, while underlined values indicate the specific group comparisons in which statistically significant differences (*p* < 0.05) were observed.

**Figure 7:**
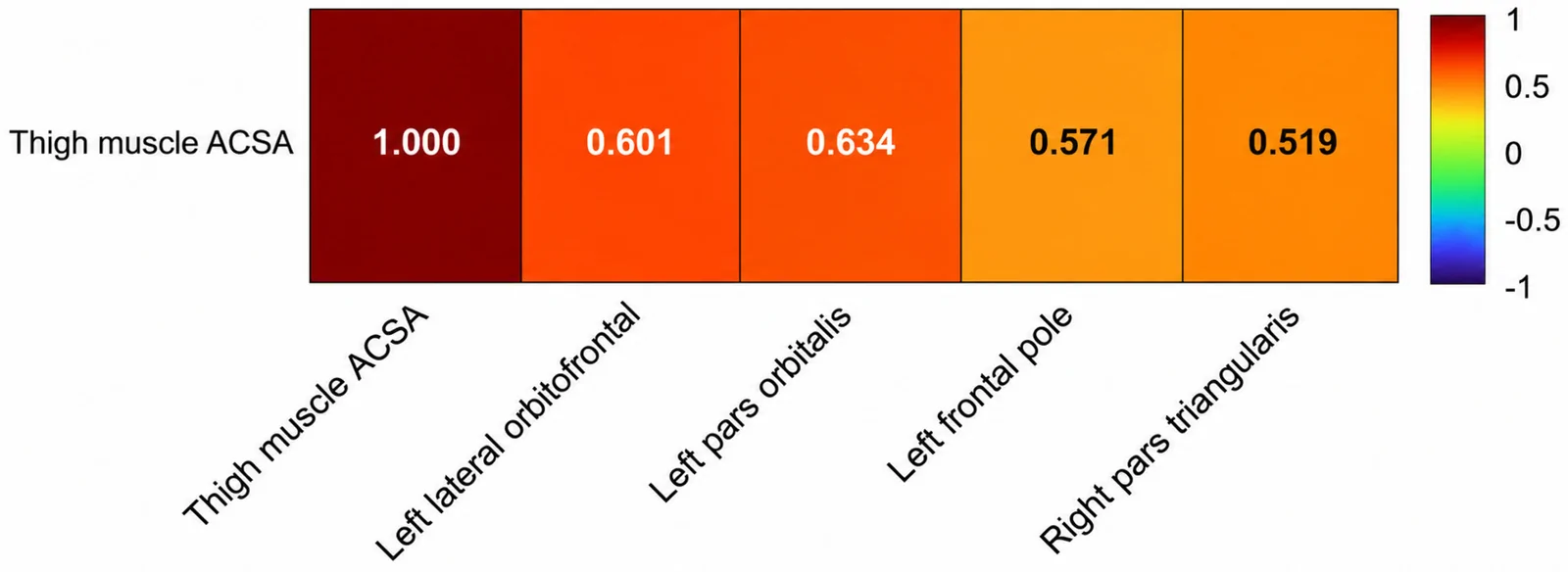
Statistically significant (*p* < 0.05) Spearman’s correlation coefficients between changes in thigh muscle ACSA and cortical thickness variables in HIRT group. Abbreviations: ACSA, anatomical cross-sectional area.

Neither HIRT, nor BFRT group showed significant positive correlation between thigh muscle ACSA and parietal cortical thickness. Instead, significant positive correlations were observed only in exploratory analyses within the HIRT group, involving the left (lateral orbitofrontal, pars opercularis, frontal pole) and right (pars triangularis) frontal lobe (Figure 7, Table 6).

**Table 6:** Exploratory Spearman’s correlations between cortical thickness changes and thigh muscle ACSA changes with corresponding BH–FDR-adjusted *q*-values in HIRT group.

Brain Region	*n*	Spearman Rho	95% CI Lower	95% CI Upper	*p* Value	BH–FDR *q*-Value
Left pars orbitalis thickness	16	0.634	0.202	0.860	0.008	0.477
Left lateral orbitofrontal thickness	16	0.601	0.150	0.845	0.014	0.477
Left frontal pole thickness	16	0.571	0.104	0.831	0.021	0.483
Right pars triangularis thickness	16	0.519	0.032	0.807	0.039	0.676

### 3.4. Regional FA Changes

**Figure 8:**
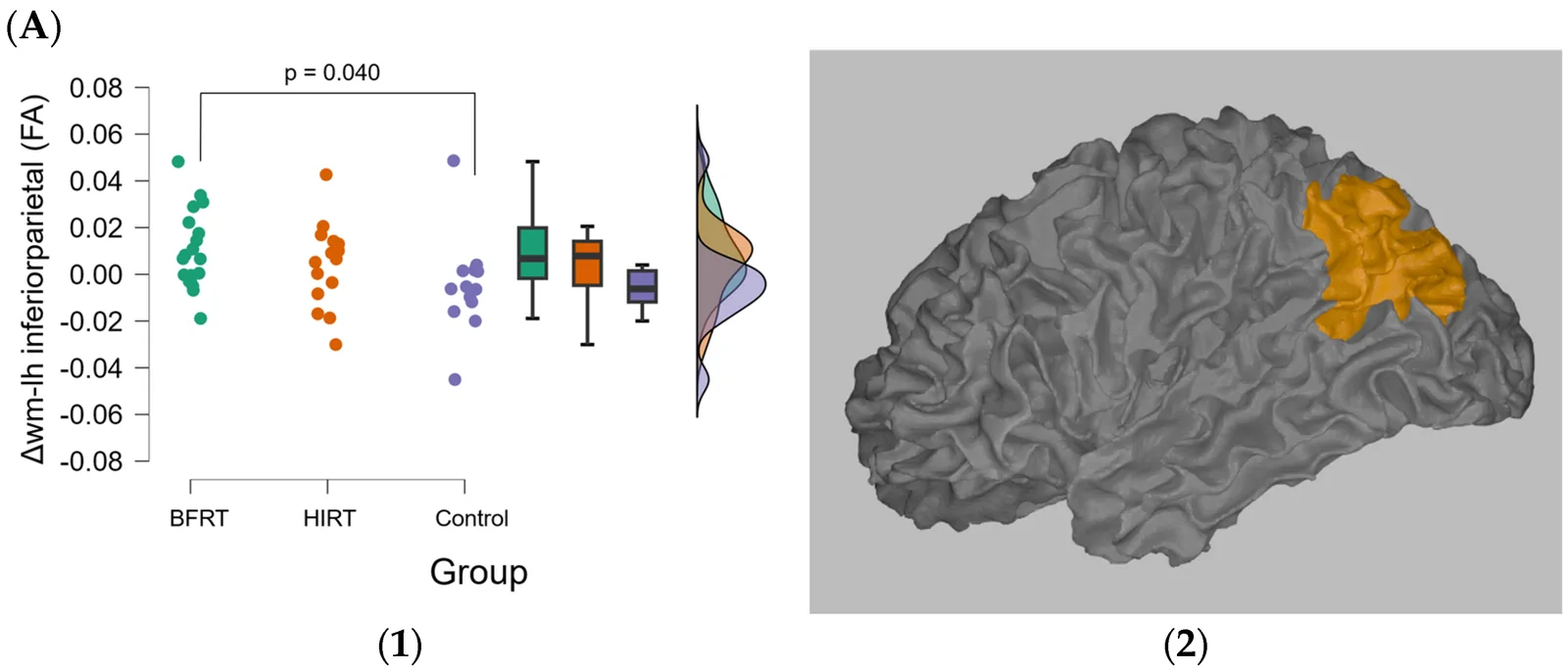

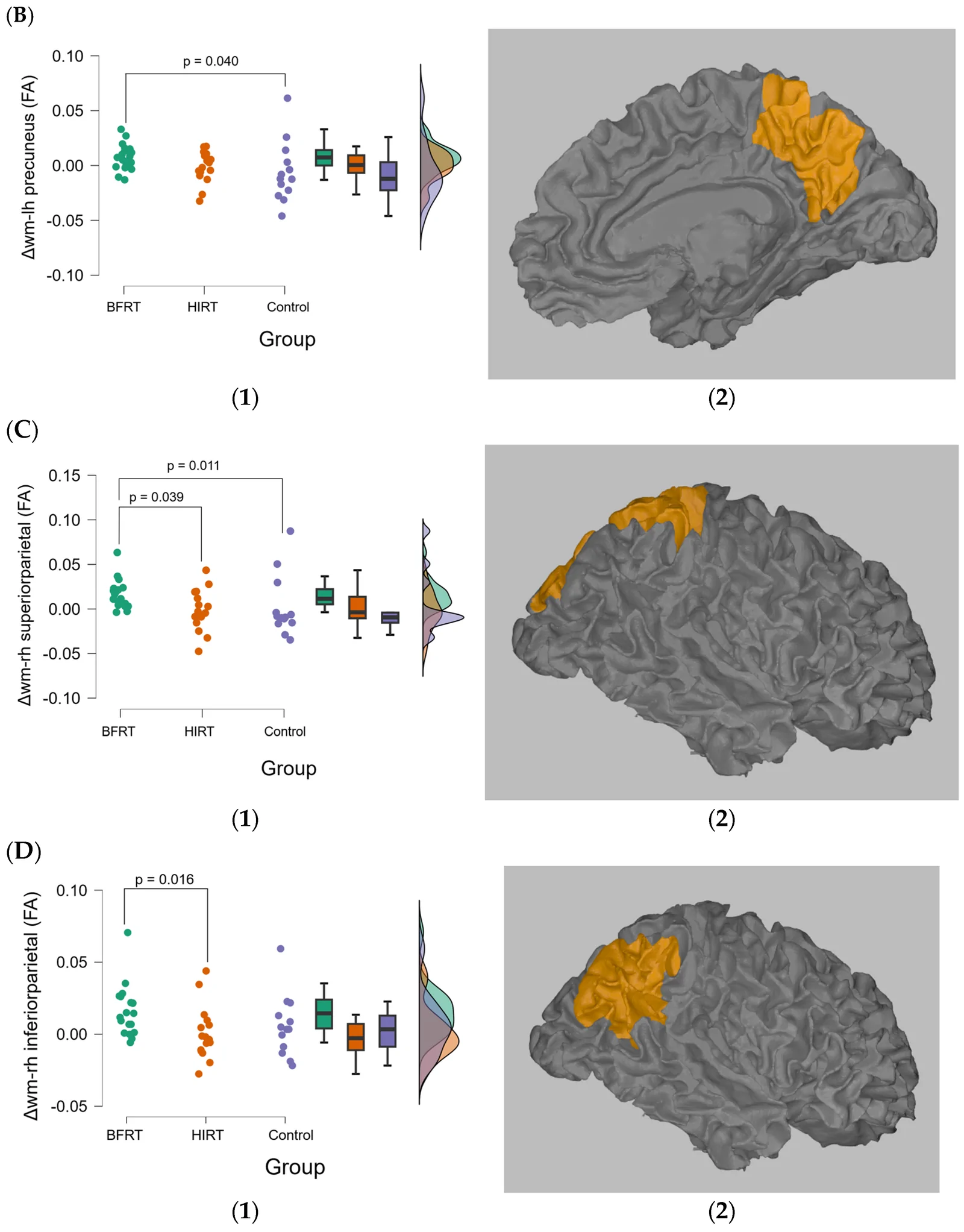
Individual data points and box plots, and violin plots (**1**) of FA value change with corresponding anatomical regions (**2**) in left inferior parietal (**A**), left precuneus (**B**), right superior parietal (**C**), right inferior parietal (**D**) regions. Individual data points represent single participants (BFRT, *n* = 17; HIRT, *n* = 16; Control, *n* = 15). Box plots display the median and interquartile range, with whiskers representing the minimum and maximum values. Violin plots illustrate the distribution density of the data. Abbreviations: lh, left; rh, right; wm, white matter; HIRT, high-intensity resistance training; BFRT, blood-flow restriction training; FA, fractional anisotropy. Brain regions are displayed on the FreeSurfer template according to the Destrieux cortical atlas; sagittal projections. Orange indicates regions demonstrating significant between-group differences. In the left hemisphere—(**A2**) left lateral view; (**B2**) left medial view. In the right hemisphere—(**C2**) right lateral view; (**D2**) right lateral view.

Table 7 summarizes the changes in FA values across all ROIs in which a significant main effect for group was observed following the 12-week intervention period (Kruskal–Wallis tests: all *p*s ≤ 0.047). Post hoc analysis revealed significant differences between the BFRT and control groups in the following parietal regions: left inferior parietal (*p* = 0.040), left precuneus (*p* = 0.040) and right superior parietal (*p* = 0.011). Moreover, significant FA value change was observed between two intervention groups, with the BFRT group demonstrating a greater increase in FA value in the right inferior (*p* = 0.016) and superior (*p* = 0.039) parietal regions. The HIRT group did not demonstrate any significant changes in FA value in the parietal region in comparison to the control group. Full results are presented in Table 7, Supplementary Materials Figure S2 and Figure 8.

**Figure 9:**
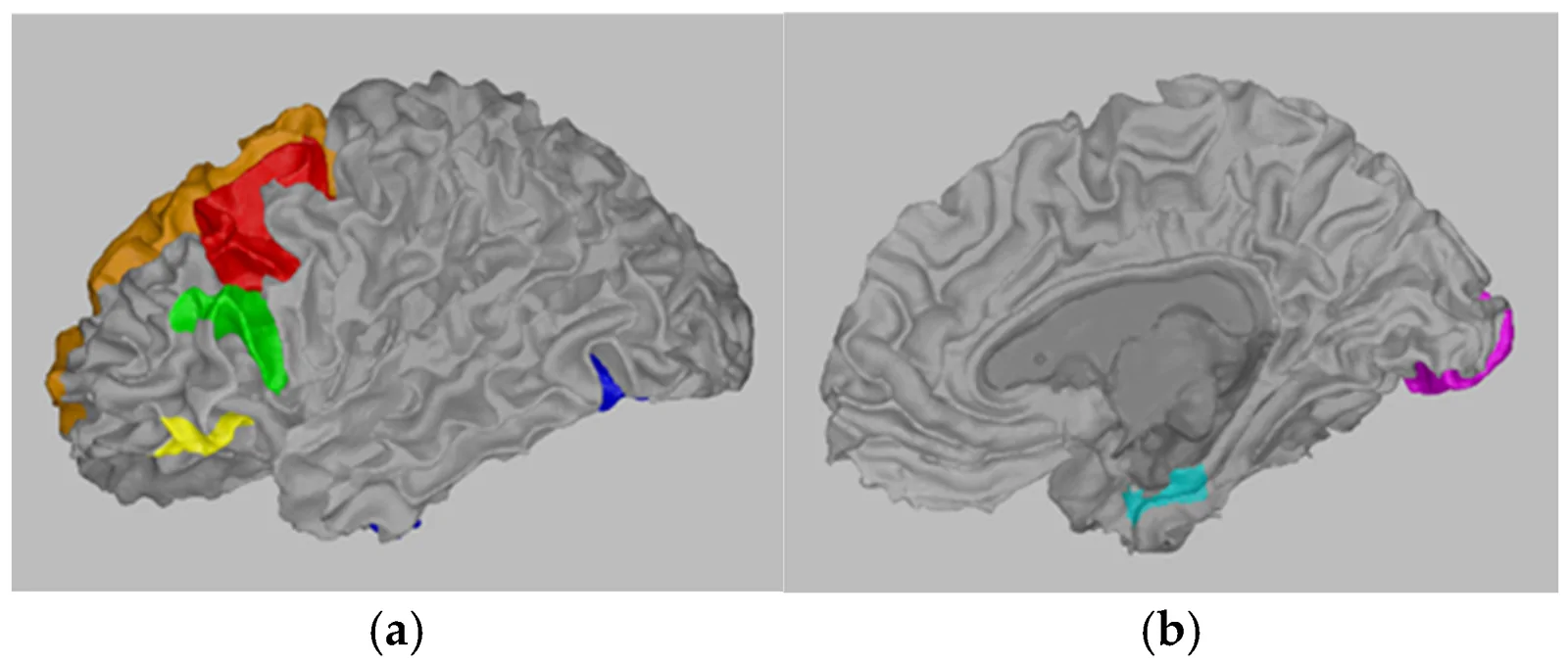
Corresponding non-parietal brain regions with significant FA change. Left hemisphere ((**a**) lateral view, (**b**) inner view), red—caudal middle frontal, orange—superior frontal, yellow—pars orbitalis, green—pars opercularis, blue—fusiform; right hemisphere, purple—lateral occipital, cyan—entorhinal. Brain regions are displayed on the FreeSurfer template according to the Destrieux cortical atlas.

Similarly, nominally significant FA changes in other regions were mainly exclusive to the BFRT group compared to controls, including these regions, left frontal (caudal middle frontal and superior frontal; both *p*s ≤ 0.039), left temporal (fusiform) and right occipital regions (lateral occipital) with both *p*s ≤ 0.027. HIRT group showed nominally statistically significant FA value increase only in the left frontal region (pars opercularis) in comparison to the control group. Additionally, the right temporal region (entorhinal) FA value was nominally significantly greater in BFRT than in the HIRT group, whereas the left frontal region (pars orbitalis) FA value was nominally significantly greater in HIRT than in BFRT group (both *p*s ≤ 0.043). Full results are presented in Table 7 and Supplementary Materials Figure S2. Corresponding brain parts are shown in Figure 9.

**Table 7:** FA changes and between-group comparisons in the three study groups.

Parietal Brain Regions	BFRT, Median (IQR)	HIRT, Median (IQR)	Control, Median (IQR)	H	df	*p*-Value	BH–FDR *q*-Value
wm-lh inferior parietal ΔFA	0.0067 (−0.0030; 0.0222)	0.0078 (0.0071; 0.0141)	−0.0052 (−0.0128; 0.0016)	6.408	2	***p*** **< 0.05 (0.041)**	Not applicable (primary analysis)
Post hoc comparisons between the groups	BFRT vs. HIRT					*p* > 0.05 (1.00)	
	BFRT vs. Control					*p* < 0.05 (0.040)	
	HIRT vs. Control					*p* > 0.05 (0.209)	
wm-lh precuneus ΔFA	0.0074 (−0.0011; 0.0150)	0.0004 (−0.0084; 0.0102)	−0.0101 (−0.0238; 0.0058)	6.337	2	***p*** **< 0.05 (0.042)**	Not applicable (primary analysis)
Post hoc comparisons between the groups	BFRT vs. HIRT					*p* > 0.05 (0.393)	
	BFRT vs. Control					*p* < 0.05 (0.040)	
	HIRT vs. Control					*p* > 0.05 (0.981)	
wm-rh superior parietal ΔFA	0.0114 (0.0043; 0.0230)	−0.0037 (−0.0138; 0.0173)	−0.0087 (−0.0157; 0.0074)	10.226	2	***p*** **< 0.05 (0.006)**	Not applicable (primary analysis)
Post hoc comparisons between the groups	BFRT vs. HIRT					*p* < 0.05 (0.039)	
	BFRT vs. Control					*p* < 0.05 (0.011)	
	HIRT vs. Control					*p* > 0.05 (1.00)	
wm-rh inferior parietal ΔFA	0.0145 (0.0012; 0.0261)	−0.0028 (−0.0115; 0.0088)	0.0034 (−0.0098; 0.0151)	8.357	2	***p*** **< 0.05 (0.015)**	Not applicable (primary analysis)
Post hoc comparisons between the groups	BFRT vs. HIRT					*p* < 0.05 (0.016)	
	BFRT vs. Control					*p* > 0.05 (0.170)	
	HIRT vs. Control					*p* > 0.05 (1.00)	
Other non-parietal brain regions							
wm-lh pars orbitalis ΔFA	−0.0071 (−0.0217; 0.0079)	0.0118 (−0.0021; 0.0294)	−0.00004 (−0.0161; 0.0149)	6.289	2	***p*** **< 0.05 (0.043)**	0.368
Post hoc comparisons between the groups	BFRT vs. HIRT					*p* < 0.05 (0.037)	
	BFRT vs. Control					*p* > 0.05 (1.00)	
	HIRT vs. Control					*p* > 0.05 (0.474)	
wm-lh pars opercularis ΔFA	0.0015 (−0.0106; 0.0086)	0.0078 (−0.0013; 0.0127)	−0.0043 (−0.0102; 0.0028)	6.125	2	***p*** **< 0.05 (0.047)**	0.368
Post hoc comparisons between the groups	BFRT vs. HIRT					*p* > 0.05 (0.426)	
	BFRT vs. Control					*p* > 0.05 (0.761)	
	HIRT vs. Control					*p* < 0.05 (0.042)	
wm-lh superior frontal ΔFA	0.0078 (0.0005; 0.0120)	0.0056 (−0.0003; 0.0122)	−0.0017 (−0.0108; 0.0042)	6.502	2	***p*** **< 0.05 (0.039)**	0.368
Post hoc comparisons between the groups	BFRT vs. HIRT					*p* > 0.05 (1.00)	
	BFRT vs. Control					*p* < 0.05 (0.046)	
	HIRT vs. Control					*p* > 0.05 (0.102)	
wm-rh entorhinal ΔFA	0.0030 (−0.0129; 0.0182)	−0.0192 (−0.0354; 0.0039)	0.00009 (−0.0217; 0.0232)	6.574	2	***p*** **< 0.05 (0.037)**	0.368
Post hoc comparisons between the groups	BFRT vs. HIRT					*p* < 0.05 (0.04)	
	BFRT vs. Control					*p* > 0.05 (1.00)	
	HIRT vs. Control					*p* > 0.05 (0.206)	
wm-rh lateral occipital ΔFA	0.0084 (−0.0007; 0.0175)	0.0005 (−0.0053; 0.0060)	−0.0008 (−0.0105; 0.0074)	7.226	2	***p*** **< 0.05 (0.027)**	0.368
Post hoc comparisons between the groups	BFRT vs. HIRT					*p* > 0.05 (0.165)	
	BFRT vs. Control					*p* < 0.05 (0.034)	
	HIRT vs. Control					*p* > 0.05 (1.00)	
wm-lh caudal middle frontal ΔFA	0.0097 (0.0010; 0.0179)	0.0141 (−0.0039; 0.0164)	−0.0075 (−0.0140; 0.0032)	7.659	2	***p*** **< 0.05 (0.022)**	0.368
Post hoc comparisons between the groups	BFRT vs. HIRT					*p* > 0.05 (1.00)	
	BFRT vs. Control					*p* < 0.05 (0.036)	
	HIRT vs. Control					*p* > 0.05 (0.056)	
wm-lh fusiform ΔFA	0.0091 (−0.0001; 0.0151)	0.0002 (−0.0074; 0.0052)	0.0002 (−0.0271; 0.0044)	8.079	2	***p*** **< 0.05 (0.018)**	0.368
Post hoc comparisons between the groups	BFRT vs. HIRT					*p* > 0.05 (0.061)	
	BFRT vs. Control					*p* < 0.05 (0.038)	
	HIRT vs. Control					*p* > 0.05 (1.00)	

Abbreviations: lh, left; rh, right; wm, white matter; FA, fractional anisotropy; HIRT, high-intensity resistance training; BFRT, blood-flow restriction training; BH–FDR, Benjamini–Hochberg false discovery rate. In the *p*-value column, bold values indicate statistically significant differences (*p* < 0.05) across all three groups, while underlined values indicate the specific group comparisons in which statistically significant differences (*p* < 0.05) were observed.

**Figure 10:**
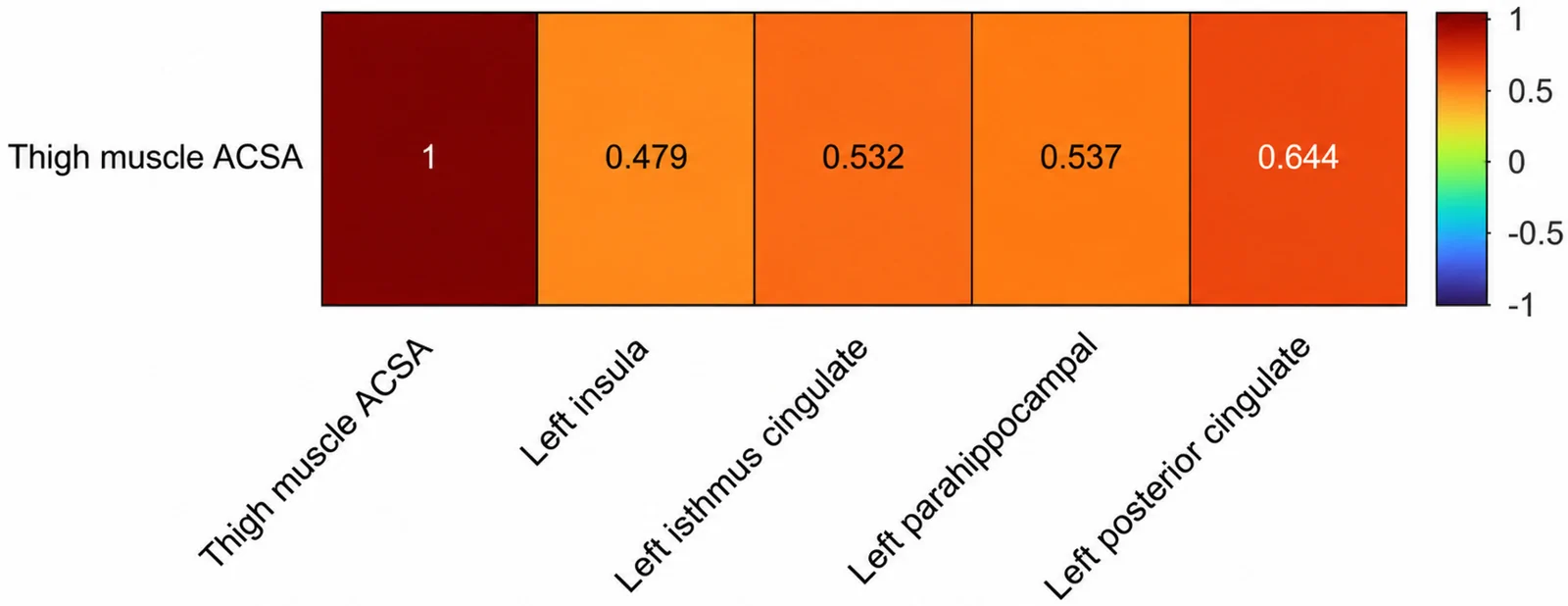
Statistically significant (*p* < 0.05) Spearman’s correlations coefficients between thigh muscle ACSA and FA value variables in BFRT group. Abbreviations: ACSA, anatomical cross-sectional area.

Significant positive correlations were observed in the BFRT group between thigh muscle ACSA and FA value in one predefined parietal region (left posterior cingulate), as well as in several additional exploratory regions in the left temporal lobe, (insula, isthmus cingulate, parahippocampal) (Figure 10, Table 8).

**Table 8:** Exploratory Spearman correlations between FA changes and thigh muscle ACSA changes with corresponding BH–FDR-adjusted *q*-values in BFRT group.

Brain Region	*n*	Spearman Rho	95% CI Lower	95% CI Upper	*p* Value	BH–FDR *q*-Value
White matter left posterior cingulate	17	0.644	0.268	0.850	0.003	Not applicable (primary analysis)
White matter left parahippocampal	17	0.537	0.109	0.797	0.018	0.434
White matter left isthmus cingulate	17	0.532	0.102	0.794	0.019	0.434
White matter left insula	17	0.479	0.032	0.766	0.038	0.517

## 4. Discussion

The primary aim of this study was to evaluate whether a lower-limb resistance training program leading to increased thigh muscle cross-sectional area and muscle mass can influence parietal cortex thickness, which was predefined as the primary hypothesis-driven region of interest. The parietal cortex is a key neural substrate involved in sensorimotor integration, proprioceptive processing, and the construction of a coherent body schema [53]; all of which are continuously engaged during lower-limb physical training [34]. Our findings did not fully support our initial hypothesis that BFRT would provide greater benefits to both muscle and brain than HIRT. HIRT demonstrated more pronounced cortical thickness changes, whereas the BFRT group showed greater increase in FA values within the predefined parietal regions, suggesting that the two exercise modalities induce distinct rather than uniformly superior patterns of neuroplastic adaptation. Moreover, exercise-induced hypertrophy of thigh muscle ACSA was associated with increased microstructural organization of FA values in the left cingulate cortex. These findings partially support the broader hypothesis that repeated engagement of these parietal functions would promote use-dependent cortical plasticity, paralleling the activity-dependent structural adaptations that underlie exercise-induced muscle hypertrophy. Although multiple anatomically related parietal measurements were evaluated, these analyses were performed within a single predefined biological hypothesis targeting exercise-related adaptations of the parietal cortex rather than as independent confirmatory tests of individual subregions. Furthermore, significant associations between gray matter neuroplastic processes and thigh muscle hypertrophy were observed only in exploratory analyses involving several prefrontal regions in the HIRT group. Because these analyses extended beyond our predefined hypothesis, they should be regarded as exploratory observations rather than confirmatory findings. Nevertheless, these observations suggest that neural adaptation to lower-limb resistance training may extend beyond the parietal cortex and may involve broader cortical networks. As the present study was designed as an exploratory pilot study, these findings should be considered hypothesis-generating and warrant confirmation in larger studies. Comparing two different resistance training programs, our study revealed that only the HIRT group showed statistically significant changes in the thickness of the left parietal cortex regions when compared with the control group, including the inferior parietal, supramarginal, and posterior cingulate cortex, whereas no corresponding effect was detected in the BFRT group. When comparing regional FA results of the parietal region between intervention groups, only the BFRT group showed significant FA changes localized to the left inferior parietal, left precuneus and right superior parietal regions. Finally, direct comparisons between the two intervention groups revealed that BFRT yielded significantly greater FA increases in the right inferior and superior parietal areas, while cortical thickness did not differ significantly between BFRT and HIRT. These findings suggest that high intensity resistance training may induce parietal cortical structural changes due to its greater mechanical loading and the associated sensorimotor integration demands, whilst BFRT, which is characterized by lower mechanical loads, may be insufficient to elicit comparable cortical adaptations. Therefore, the findings do not support the initial hypothesis that BFRT is universally superior to HIRT for promoting structural brain adaptations. Notably, comparisons with previous resistance training studies remain challenging as the existing knowledge on resistance training-induced structural brain changes is limited and has not yet been systematically synthesized [54]. Furthermore, direct evidence documenting resistance training-induced changes in specific prefrontal, temporal and parietal subregions remain scarce. Despite these limitations, previous studies have investigated functional brain changes associated with resistance exercises [55], with research suggesting that such exercise is associated with slower white matter degradation and more advantageous brain hemodynamics, which may in turn support functional improvements in cognition, especially in patients with Alzheimer‘s disease. It should be noted, however, that the aforementioned study focused primarily on brain blood flow, oxygenation and neuroinflammation, rather than brain volume or mass. In a related line of research, a separate study examined the effects of multimodal exercise on cerebral blood flow in patients who were at least three months post-stroke. The investigators of this study reported that the strongest regional changes in blood flow were observed in the parietal lobe after six months of combined training [56]. Collectively, these findings highlight that direct comparisons with the existing literature remain limited, as no prior exercise studies have specifically examined the effects of resistance training on parietal lobe thickness or white matter integrity.

To extend our analysis, we investigated whether the two resistance exercise modalities induce comparable structural brain adaptations beyond the primary hypothesis-driven region of interest, namely the parietal cortex. Although none of the exploratory findings remained statistically significant after BH-FDR correction and should be regarded as hypothesis-generating, several nominally significant associations clustered across anatomically related regions. This pattern may indicate the presence of a weak underlying biological signal that could not be confirmed in the present pilot study because of the limited sample size. Therefore, these observations require conformation in larger cohorts.

Exploratory analyses suggested that consistent with the findings observed in the parietal region, only the HIRT group demonstrated nominal between-group differences in several left non-parietal cortical regions compared with the control group, including subregions of the temporal cortex (bankssts, inferior temporal), frontal cortex (medial orbitofrontal, precentral, rostral middle frontal), and occipital cortex (lateral occipital). However, these findings should be interpreted with caution, as they were identified in exploratory analyses and do not provide definitive evidence of exercise-induced cortical adaptations. Rather, they represent hypothesis-generating observations that may help identify potential brain regions for future investigation in larger studies.

When comparing the two intervention groups, exploratory analyses suggested a greater increase in left medial orbitofrontal thickness and left rostral middle frontal thickness in the HIRT group compared to the BFRT group. However, these findings should be interpreted with caution, as they originated from exploratory analyses and do not provide definitive evidence of different intervention effects. One possible explanation is that HIRT imposes greater central motor drive and executive control demands. As previously noted, only a limited number of studies have directly investigated cortical thickness changes after resistance training alone [37], with most of the existing research focusing on the neuroplastic effects of aerobic or combined exercise modalities [57,58]. The findings, however, remain inconsistent across intervention modalities and subject groups. Bashir et al. [57] examined exercise-induced cortical changes following six months of aerobic and anaerobic physical exercise, reporting significantly increased gray matter thickness predominantly in the occipital, frontal and parietal lobes. In contrast, Kušleikienė et al. [37] found that 12 weeks of moderate-to-vigorous resistance training targeting lower-limb musculature induced a protective effect on gray matter structures in the prefrontal and temporal lobes; however, between-group differences in pre-to-post changes in cortical thickness did not reach statistical significance. The more pronounced cortical thickness increases in the HIRT group raise the possibility that exercise intensity is an important modulator of gray matter neuroplasticity. These findings contrast with our initial expectation that BFRT would produce greater structural brain adaptations, suggesting that higher mechanical loading may be more relevant for cortical remodeling than initially hypothesized. Together, these findings suggest that exercise intensity and intervention duration may be key determinants of gray matter neuroplasticity, highlighting the need for future studies to investigate their dose–response relationship with cortical structural change.

In addition to cortical thickness, we further investigated brain white matter microstructure by analyzing FA value changes associated with the two resistance training modalities. Within our primary hypothesis-driven region of interest, the parietal lobe, only the BFRT group demonstrated significant FA value change compared to the controls, contrasting with the cortical findings observed in both groups. Corresponding parietal regions exhibiting these changes included the left inferior parietal, left precuneus and right superior parietal cortices. Furthermore, significant differences in FA value were observed between the two intervention groups, with greater increase in FA observed in the right inferior and superior parietal regions in the BFRT group compared to HIRT. These observations suggest that BFRT may preferentially influence parietal white matter microstructure through metabolic or hemodynamic mechanisms, that are distinct from the cortical structural adaptations associated with HIRT. Beyond the predefined parietal region of interest, exploratory analyses identified nominal between-group differences in FA within additional white matter regions. Specifically, the BFRT group showed nominal increases in FA value in comparison to the control group in the left frontal (caudal middle frontal and superior frontal), left temporal (fusiform) and right occipital regions (lateral occipital), while HIRT group showed FA value increase only in the left frontal region (pars opercularis) in comparison to control group. As mentioned earlier, these findings should be interpreted with caution, as they originated from exploratory analyses and therefore represent hypothesis-generating observations that require confirmation in larger studies. However, evidence on the effects of resistance training on white matter microstructural integrity remains limited; therefore, it remains challenging to establish whether the two exercise modalities selectively drive the gray versus white matter adaptations observed in our study cohort. Notably, traditional resistance training has demonstrated measurable effects on white matter integrity, with emerging evidence that highlights its structural brain benefits. For example, Oh et al. reported that a twelve-month resistance training program significantly increased white matter integrity index as shown by the higher T1W/T2W ratio compared to controls in the external capsule and posterior thalamic radiations [59]. Similarly, Stephen et al., 2020 [60] demonstrated that two years of combining aerobic and resistance training in an older Finnish cohort elicited longitudinal changes in DTI parameters of the corpus callosum, optic radiation, posterior cingulate, internal/external capsules and frontal white matter tracts. Collectively, these findings with our primary hypothesis-driven results suggest that exercise-induced improvements in white matter microstructure may represent a key neurobiological mechanism underlying the cognitive benefits of resistance training in older adults; the exploratory observations reported in the present study require confirmation before firm conclusions regarding white matter adaptations can be drawn. However, no studies have directly examined the potential beneficial effects of BFRT on white matter microstructural organization using DTI-derived neuroimaging biomarkers, nor have they elucidated the underlying molecular and cellular signaling pathways through which such adaptations may occur.

The observed difference in parietal cortex thickness between the two intervention groups could be explained by evidence suggesting that high-intensity resistance training demands greater sensorimotor integration and proprioceptive processing functions for which the parietal cortex is primarily responsible [61]. Additionally, high-load resistance training has been linked to increases in Brain-derived neurotrophic factor (BDNF) and insulin-like growth factor 1, which support synaptogenesis and dendritic growth [62]. However, these mechanisms were not directly evaluated in our study and therefore should be considered hypothetical. Consequently, the greater cortical thickness changes in the HIRT group may be related to higher sensorimotor and mechanical demands imposed by high-intensity resistance training rather than to a specific neurotrophic mechanism. However, within the BFRT group, in which physiological stress is expected to be overregulated by partial vascular occlusion, we observed higher FA values in DTI data compared to the HIRT group. In our study, lactate concentrations increased significantly following the first BFRT, whereas CK, a biomarker of exercise-induced muscle damage, showed a greater, but non-significant increase following HIRT. These findings are consistent with the proposed physiological differences between two exercise modalities. One possible explanation of this finding could be explained by the hypothesis that low-load training with partial blood-flow restriction may reduce mechanical strain and generate a milder systemic stress response than HIRT, which may contribute to the distinct patterns of white matter adaptation observed in the present study. Furthermore, BFRT may induce a hypoxic environment that has been hypothesized to promote angiogenesis, increase release of growth factors such as vascular endothelial growth factor and possibly BDNF, which in turn may support myelin maintenance and repair [60]. These mechanisms could contribute to more organized white matter microstructure which may partially account for the higher FA values observed in the BFRT group at the end of intervention. Beyond the aforementioned mechanistic explanations, converging evidence from other studies suggests that resistance training may increase circulating myokines levels which may exert positive effects on brain health through neurotrophic, metabolic and anti-inflammatory pathways [63]. However, these biomarkers were not measured in the present study and should be interpreted as hypothesis rather than demonstrated effects.

Our study has several limitations. This study was designed as an exploratory pilot study; therefore, no formal a priori sample size calculation was performed. The findings should be considered preliminary and require confirmation in larger longitudinal studies. The allocation of participants into three study groups resulted in a relatively small number of participants in each group, which likely reduced the statistical power needed to detect subtle between-group differences and ultimately required the use of non-parametric tests for between-group analyses. In addition, participant drop-out further contributed to this reduced sample size. Although drop-out rates were moderate (up to six participants in the control group and fewer in the intervention groups), this is a well-recognized limitation of longitudinal intervention studies. As drop-outs only had baseline measures, estimation of treatment effects was considered to be based too much on assumptions. Hence, no intention-to-treat analysis was applied. Accordingly, the analysis was based on a complete-case approach. Participants without post-intervention MRI examinations were excluded, which may have introduced healthy completer bias. Because the primary outcomes were MRI-derived structural measures, missing data was not imputed. Although the parietal cortex was predefined as the primary region of interest, analyses of the remaining brain regions were exploratory and involved multiple statistical comparisons. While correction for multiple testing was applied to these exploratory analyses, the possibility of both false-positive and false-negative findings cannot be excluded. Therefore, the exploratory findings should be interpreted with caution rather than as confirmatory. Furthermore, no comprehensive cognitive or functional outcomes were integrated into the present analyses. The clinical relevance of the observed changes remains to be established. In addition, only immediate post-intervention outcomes were assessed. The absence of long-term follow-up precludes conclusions regarding the persistence of the observed structural brain adaptations. MRI-derived measures of cortical thickness and white matter microstructure are inherently subject to measurement variability despite standardized acquisition, quality-control procedures, and visual inspections of datasets. In addition, no mechanistic biomarkers (such as neurotrophic, inflammatory, angiogenic, metabolic biomarkers) were measured. Therefore, the biological mechanisms underlying the observed findings remain speculative and cannot be directly inferred from the present study. Moreover, FA is an indirect marker of white matter microstructure and may be influenced by multiple biological factors, including axonal organization, myelination, fiber density and crossing fibers. Therefore, changes in FA should not be interpreted as direct evidence of specific microstructural alterations. Furthermore, the differences in muscle mass gain following resistance exercise training could have been influenced by differences in nutrient (in particular protein) intake among participants. It is a limitation of this study that participants were not guided in their protein intake and participants’ protein intake was not monitored. Finally, only men were recruited to the study, which may have limited the external validity of the findings to the general population. Further investigations incorporating women would be beneficial in order to evaluate the observed effects across a broader population.

## 5. Conclusions

To conclude, our observation suggests that 12 weeks of both HIRT and BFRT methods are associated with beneficial structural changes, occurring via distinct pathways. HIRT-related changes were primarily observed as positive changes in cortical thickness, whereas BFRT-related adaptations were primarily evident in beneficial alterations of brain microstructure. These findings may have some clinical implications, suggesting that individuals with higher physical capacity may benefit more from HIRT in preserving cortical thickness, which is particularly relevant in an aging population given the associated cortical atrophy. Thus, BFRT may represent a promising alternative for older adults or those with limited physical capacity, by preserving structural connectivity. The observed MRI findings should be considered exploratory and future studies with larger sample sizes, inclusion of women, and extended intervention periods are needed to further increase our understanding of the beneficial effects of resistance training on brain structure and microstructure.

## Data Availability

The data supporting the conclusions of this article will be made available by the authors on reasonable request due to technical and privacy reasons.

## Supplementary Materials

The following supporting information can be downloaded at https://www.mdpi.com/article/10.3390/brainsci16080797/s1, Figure S1: Individual data points and box plots, and violin plots (1) of the change in cortical thickness with corresponding anatomical regions (2) in left bankssts cortex (A), left inferior temporal cortex (B), left medial orbitofrontal cortex (C), left precentral cortex (D), left rostral middle frontal (E) and left lateral occipital cortex (F); Figure S2: Individual data points and box plots (1) of FA value change with corresponding anatomical regions (2) in left caudal middle frontal (A), left superior frontal (B), left pars orbitalis (C), left pars opercularis (D), left fusiform (E), right lateral occipital (F) and right entorhinal (G) regions.

## Abbreviations

The following abbreviations are used in this manuscript:

MRI	Magnetic resonance imaging
HIRT	High-intensity resistance training
BFRT	Blood-flow restriction training
ACSA	Anatomical cross-sectional area
CK	Creatine kinase
BRAIN-M	Blood-Flow Restriction and High-Intense Resistance Training in Aging: Interactions Between Neuroplasticity and Muscle
BMI	Body mass index
RPE	Rate of perceived exertion
IPAQ-SF	International Physical Activity Questionnaire—Short Form
RM	Repetition maximum
DTI	Diffusion tensor imaging
FA	Fractional anisotropy
RF	Rectus femoris
VL	Vastus lateralis
VI	Vastus intermedius
VM	Vastus medialis
BFS	Biceps femoris short head
BFL	Biceps femoris long head
ST	Semitendinosus
SM	Semimembranosus
AD	Adductors
GR	Gracilis
SR	Sartorius
ALOP	Arterial limb occlusion pressure
EOE	Eccentric-only exercise
RT	Resistance training
BH-FDR	Benjamini–Hochberg false discovery rate
ROI	Region of interest
BDNF	Brain-derived neurotrophic factor
