# Supplementary material for: Differential Effects of Blood-Flow Restriction and High-Intensity Resistance Training on Cortical Thickness and White Matter Integrity in Older Men: A Three-Group Randomized Controlled Trial
Source: Brain Sci. 2026 Jul 28;16(8):797. doi: 10.3390/brainsci16080797 (PMC13511528; doi:10.3390/brainsci16080797)
Supplement: Supplementary file 1 [file brainsci-16-00797-s001.zip › Figure S2.pdf]

A

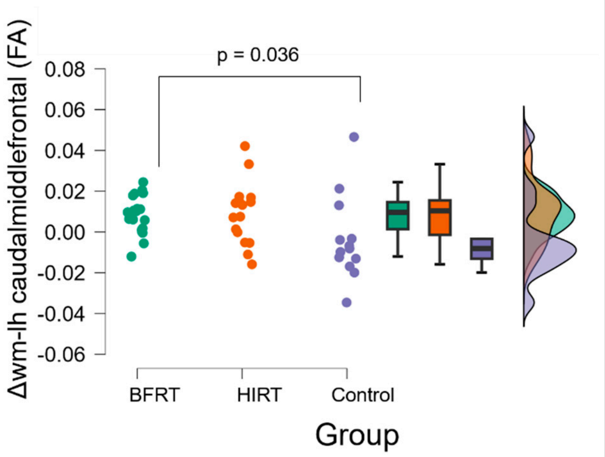
p = 0.036
0.08
0.06
0.04
0.02
0.00
-0.02
-0.04
-0.06
Δwm-lh caudalmiddlefrontal (FA)
BFRT
HIRT
Control
Group

(1)

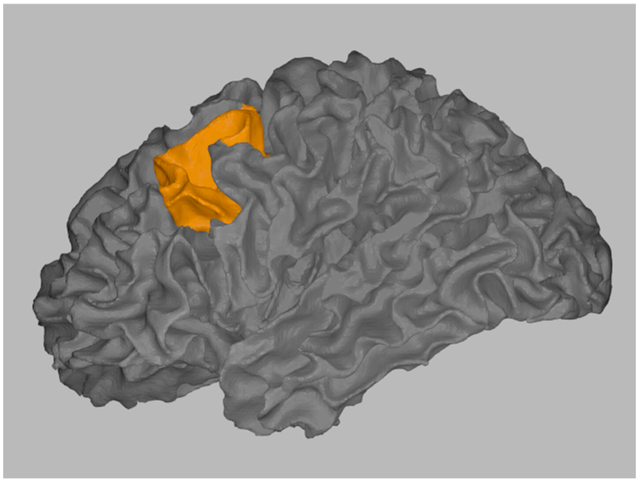

(2)

B

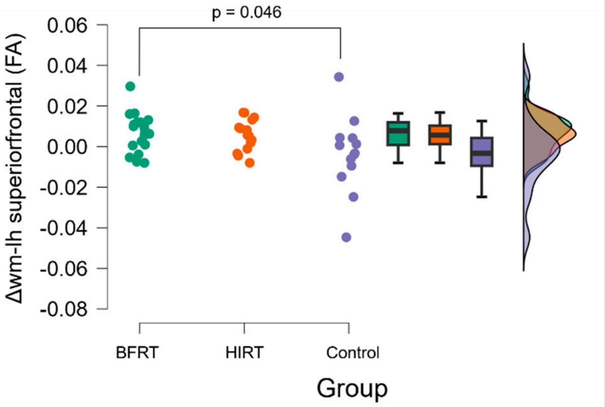
p = 0.046
0.06
0.04
0.02
0.00
-0.02
-0.04
-0.06
-0.08
Δwm-lh superiorfrontal (FA)
BFRT
HIRT
Control
Group

(1)

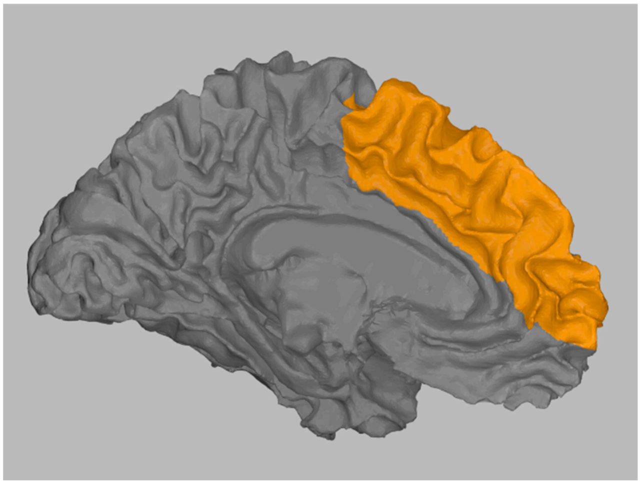

(2)

C

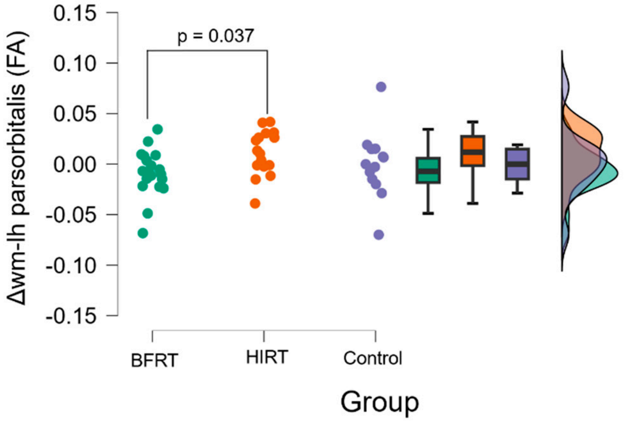
0.15
0.10
0.05
0.00
-0.05
-0.10
-0.15
Δwm-lh parsorbitalis (FA)
p = 0.037
BFRT
HIRT
Control
Group

(1)

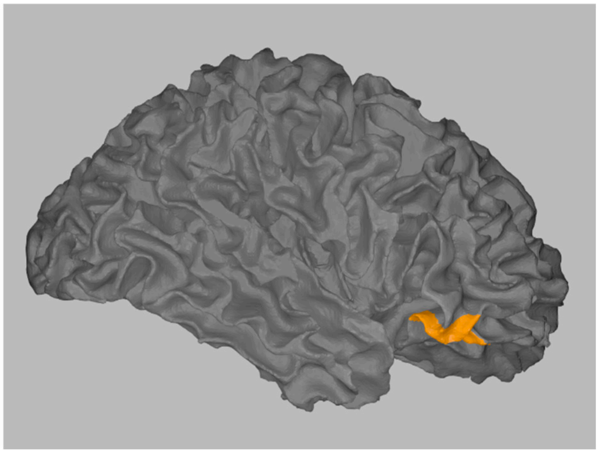

(2)

D

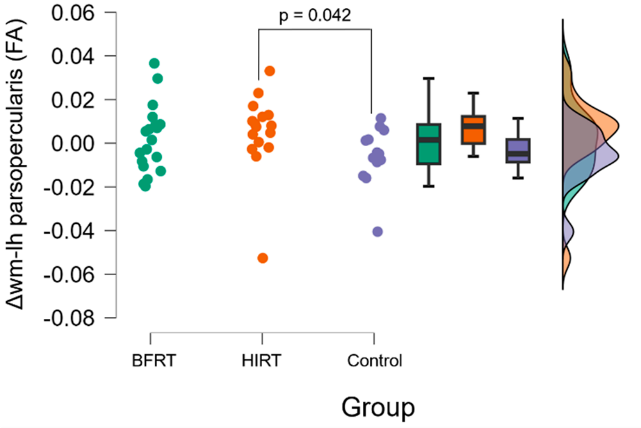
0.06
0.04
0.02
0.00
-0.02
-0.04
-0.06
-0.08
Δwm-lh parsopercularis (FA)
p = 0.042
BFRT
HIRT
Control
Group

(1)

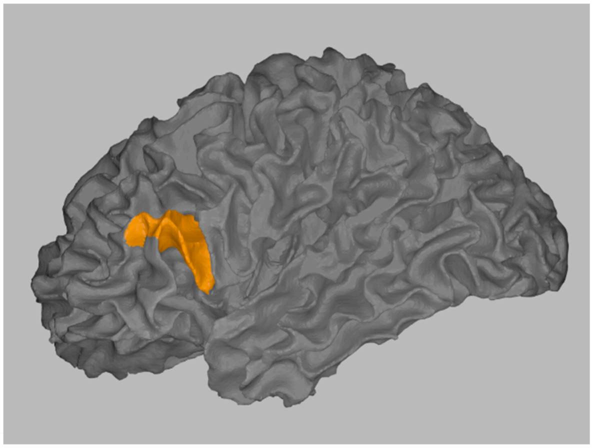

(2)

E

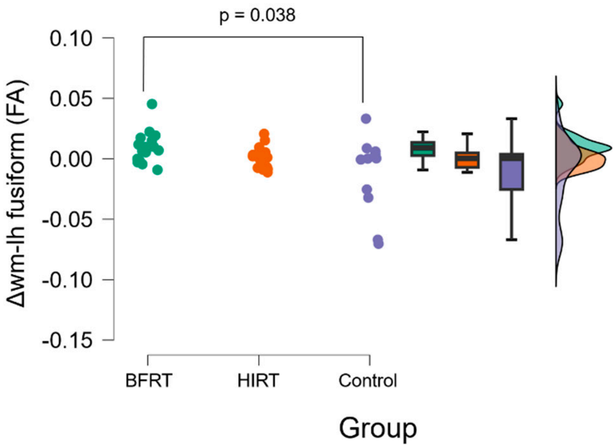
p = 0.038
0.10
0.05
0.00
-0.05
-0.10
-0.15
Δwm-lh fusiform (FA)
BFRT
HIRT
Control
Group

(1)

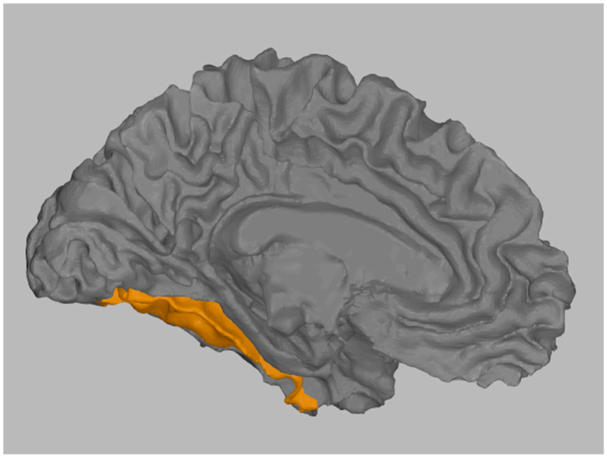

(2)

F

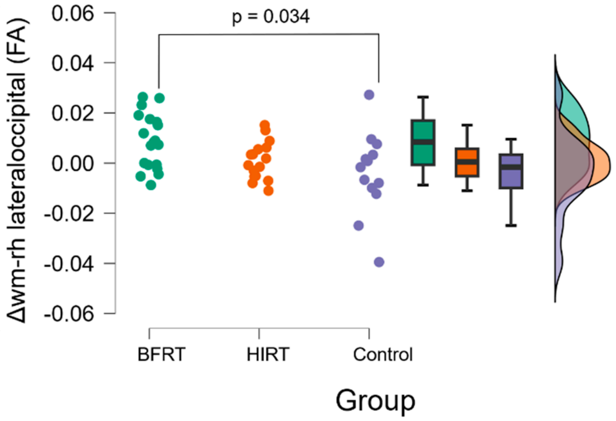
p = 0.034
0.06
0.04
0.02
0.00
-0.02
-0.04
-0.06
Δwm-rh lateraloccipital (FA)
BFRT
HIRT
Control
Group

(1)

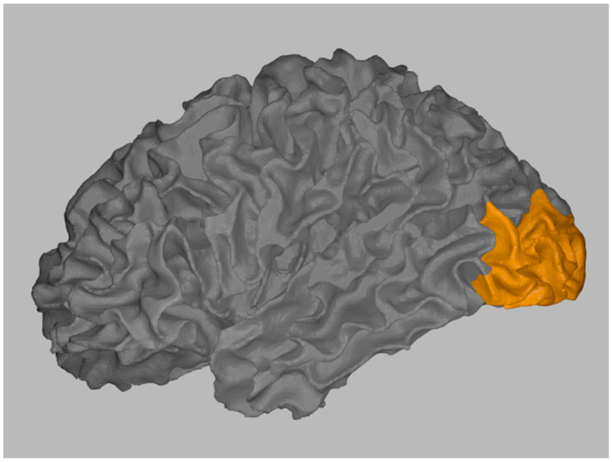

(2)

G

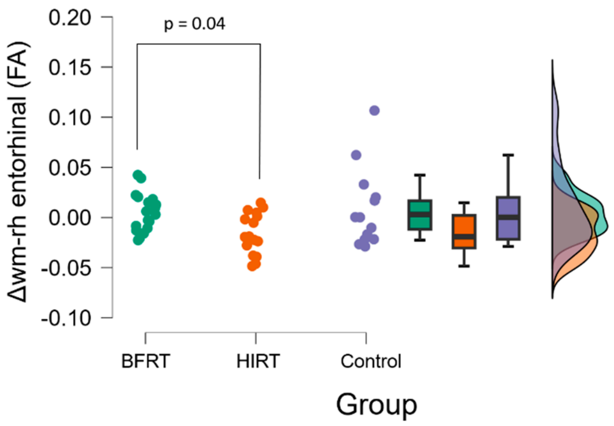

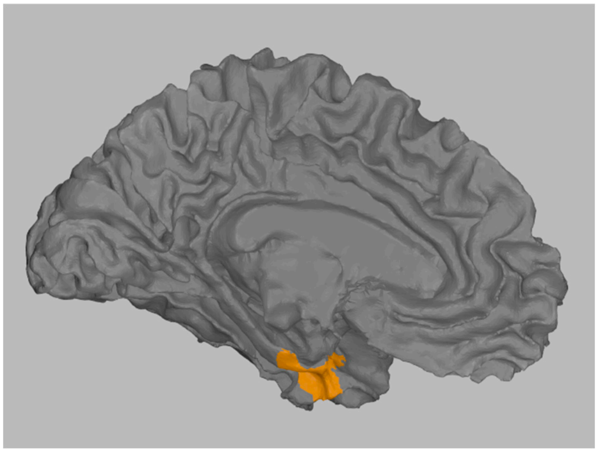

(1) **(2)**

Figure S2. Individual data points and box plots (1) of FA value change with corresponding anatomical regions (2) in left caudal middle frontal (A), left superior frontal (B), left pars orbitalis (C), left pars opercularis (D), left fusiform (E), right lateral occipital (F) and right entorhinal (G) regions. Individual data points represent single participants (BFRT, n = 17; HIRT, n = 16; Control, n = 15). Box plots display the median and interquartile range, with whiskers representing the minimum and maximum values. Violin plots illustrate the distribution density of the data. Abbreviations: lh, left; rh, right; wm, white matter; HIRT, high-intensity resistance training; BFRT, blood-flow restriction training. Brain regions are displayed on the FreeSurfer template according to the Destrieux cortical atlas; sagittal projections. Orange indicates regions demonstrating significant between-group differences. Left hemisphere— A (2) left lateral view; B (2) left medial view; C (2) left lateral view; D (2) left lateral view; E (2) left lateral view. Right hemisphere— F (2) right lateral view; G (2) right medial view.
