# Supplementary material for: Differential Effects of Blood-Flow Restriction and High-Intensity Resistance Training on Cortical Thickness and White Matter Integrity in Older Men: A Three-Group Randomized Controlled Trial
Source: Brain Sci. 2026 Jul 28;16(8):797. doi: 10.3390/brainsci16080797 (PMC13511528; doi:10.3390/brainsci16080797)
Supplement: Supplementary file 1 [file brainsci-16-00797-s001.zip › Figure S1.pdf]

A

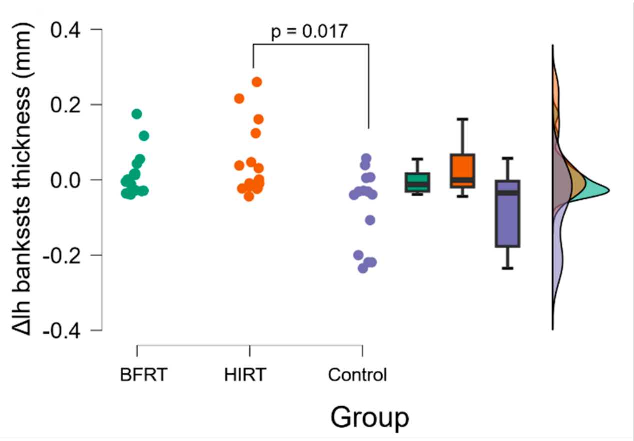
p = 0.017
Δlh bankssts thickness (mm)
0.4
0.2
0.0
-0.2
-0.4
BFRT
HIRT
Control
Group

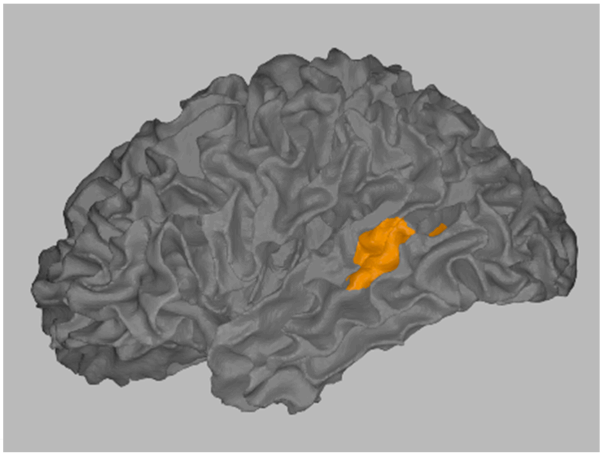

(1) **(2)**

B

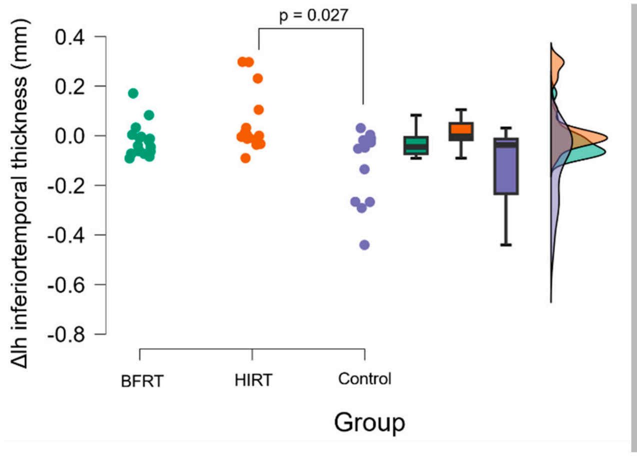
p = 0.027
Δlh inferiortemporal thickness (mm)
0.4
0.2
0.0
-0.2
-0.4
-0.6
-0.8
BFRT
HIRT
Control
Group

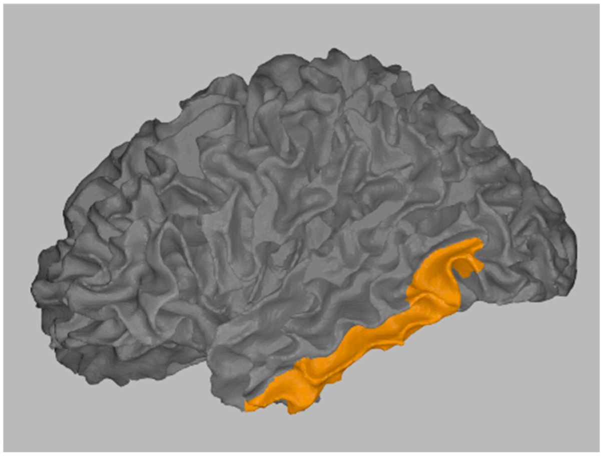

(1) **(2)**

C

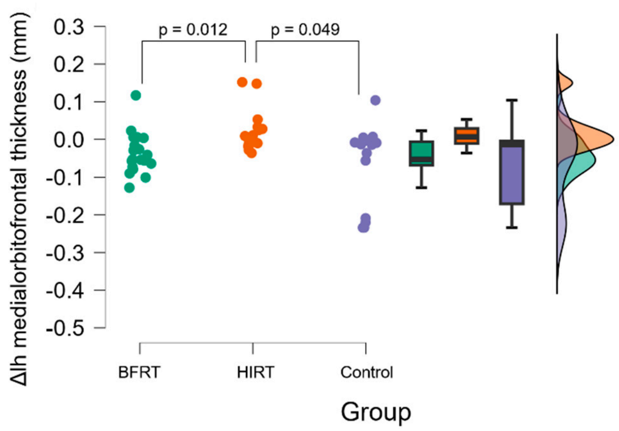

Δlh medialorbitofrontal thickness (mm)
0.3
0.2
0.1
0.0
-0.1
-0.2
-0.3
-0.4
-0.5
p = 0.012
p = 0.049
BFRT
HIRT
Control
Group

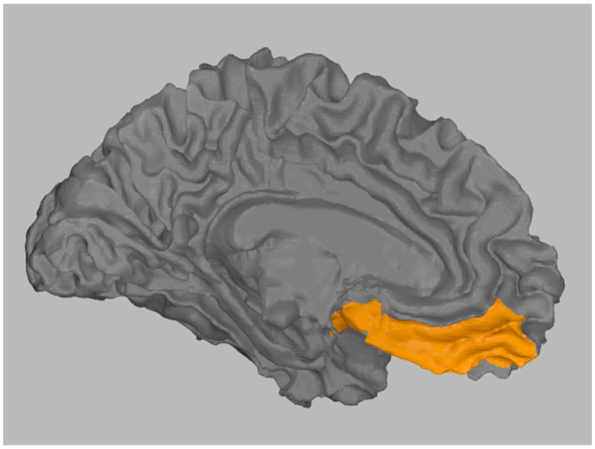

(1) **(2)**

D

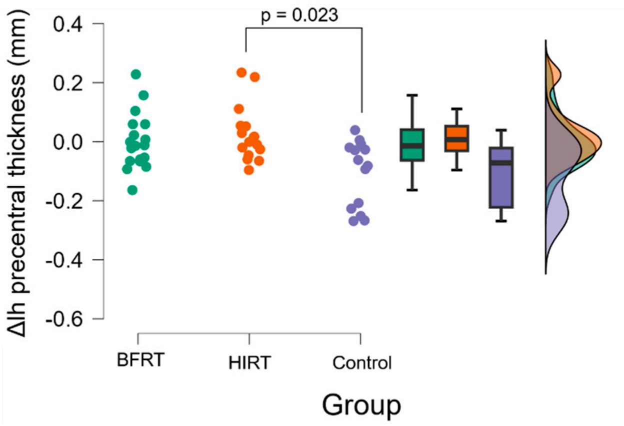

Δlh precentral thickness (mm)
0.4
0.2
0.0
-0.2
-0.4
-0.6
p = 0.023
BFRT
HIRT
Control
Group

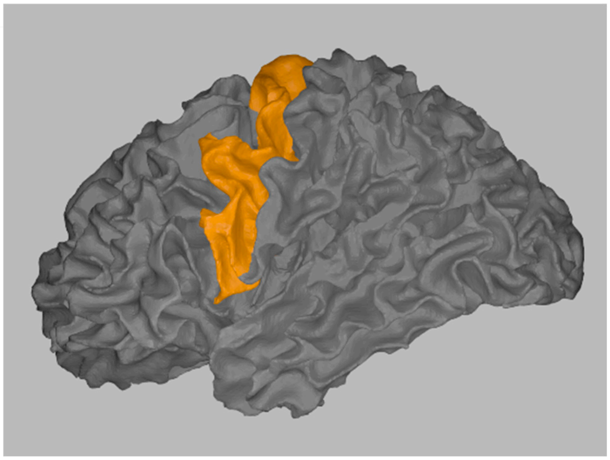

(1) **(2)**

E

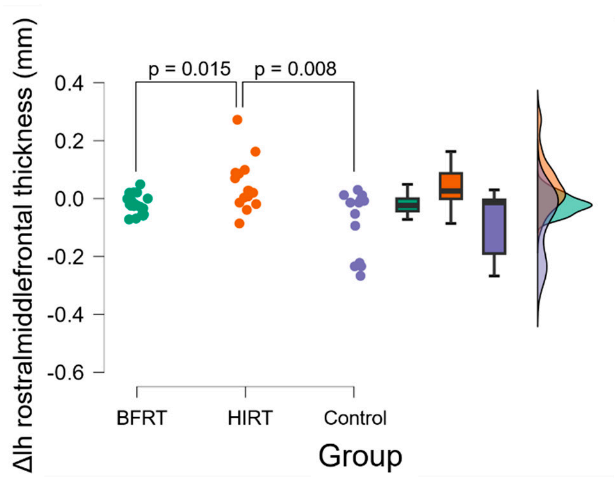

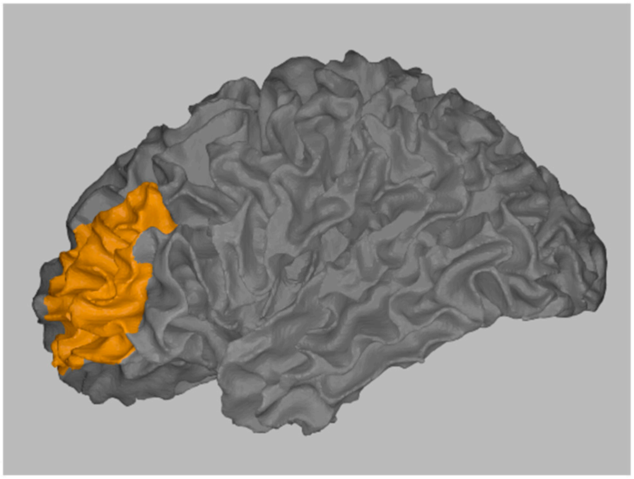

(1) **(2)**

F

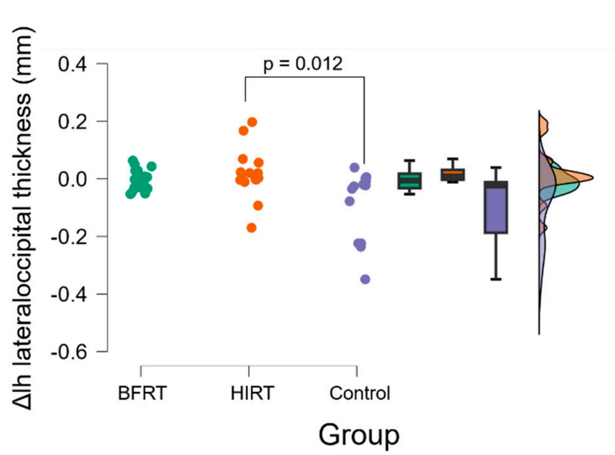

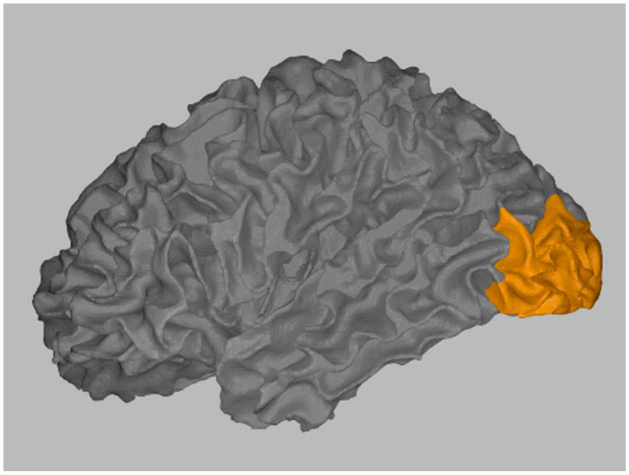

(1) **(2)**

Figure S1. Individual data points and box plots, and violin plots (1) of the change in cortical thickness with corresponding anatomical regions (2) in left bankssts cortex (A), left inferior temporal cortex (B), left medial orbitofrontal cortex (C), left precentral cortex (D), left rostral middle frontal (E) and left lateral occipital cortex (F). Individual data points represent single participants (BFRT, n = 17; HIRT, n = 16; Control, n = 15). Box plots display the median and interquartile range, with whiskers representing the minimum and maximum values. Violin plots illustrate the distribution density of the data. Abbreviations: lh, left; HIRT, high-intensity resistance training; BFRT, blood-flow restriction training. Brain regions are displayed on the FreeSurfer template according to the Destrieux cortical atlas; sagittal projections; regions in the left hemisphere are displayed. Orange indicates regions demonstrating significant between-group differences. A (2) left lateral view; B (2) left lateral view; C (2) left medial view; D (2) left lateral view; E (2) left lateral view; F (2) left lateral view.
